# Ultra‐high sensitivity mass spectrometry quantifies single‐cell proteome changes upon perturbation

**DOI:** 10.15252/msb.202110798

**Published:** 2022-02-28

**Authors:** Andreas‐David Brunner, Marvin Thielert, Catherine Vasilopoulou, Constantin Ammar, Fabian Coscia, Andreas Mund, Ole B Hoerning, Nicolai Bache, Amalia Apalategui, Markus Lubeck, Sabrina Richter, David S Fischer, Oliver Raether, Melvin A Park, Florian Meier, Fabian J Theis, Matthias Mann

**Affiliations:** ^1^ Proteomics and Signal Transduction Max‐Planck Institute of Biochemistry Martinsried Germany; ^2^ NNF Center for Protein Research Faculty of Health and Medical Sciences University of Copenhagen Copenhagen Denmark; ^3^ EvoSep Biosystems Odense Denmark; ^4^ Bruker Daltonik GmbH Bremen Germany; ^5^ Helmholtz Zentrum München – German Research Center for Environmental Health Institute of Computational Biology Neuherberg Germany; ^6^ TUM School of Life Sciences Weihenstephan Technical University of Munich Freising Germany; ^7^ Bruker Daltonics Inc. Billerica MA USA; ^8^ Functional Proteomics Jena University Hospital Jena Germany

**Keywords:** drug perturbation, low‐flow LC–MS, proteomics at single‐cell resolution, single‐cell heterogeneity, systems biology, Cell Cycle, Proteomics

## Abstract

Single‐cell technologies are revolutionizing biology but are today mainly limited to imaging and deep sequencing. However, proteins are the main drivers of cellular function and in‐depth characterization of individual cells by mass spectrometry (MS)‐based proteomics would thus be highly valuable and complementary. Here, we develop a robust workflow combining miniaturized sample preparation, very low flow‐rate chromatography, and a novel trapped ion mobility mass spectrometer, resulting in a more than 10‐fold improved sensitivity. We precisely and robustly quantify proteomes and their changes in single, FACS‐isolated cells. Arresting cells at defined stages of the cell cycle by drug treatment retrieves expected key regulators. Furthermore, it highlights potential novel ones and allows cell phase prediction. Comparing the variability in more than 430 single‐cell proteomes to transcriptome data revealed a stable‐core proteome despite perturbation, while the transcriptome appears stochastic. Our technology can readily be applied to ultra‐high sensitivity analyses of tissue material, posttranslational modifications, and small molecule studies from small cell counts to gain unprecedented insights into cellular heterogeneity in health and disease.

## Graphical abstract

A new ultra‐high sensitivity LC‐MS workflow increases sensitivity by up to two orders of magnitude and enables true single‐cell proteome analysis. In‐depth comparison indicates that the single‐cell transcriptome is stochastic while the single‐cell proteome is complete and stable.

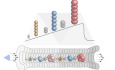

## Introduction

In single‐cell analysis, biological variability can directly be attributed to individual cells instead of being averaged over an ensemble or complex tissue (Regev *et al*, 2017). While microscopy has always been single‐cell based, specialized deep sequencing technologies have achieved this for systems biological approaches (Smith *et al*, 2010; Ramsköld *et al*, 2012; Jaitin *et al*, 2014; Schnitzbauer *et al*, 2017; Schaum *et al*, 2018; Lundberg & Borner, 2019). At the level of proteins, the functional actors of cells, single cells are currently studied by antibody‐based technologies, which are by necessity directed against previously chosen targets (Uhlén *et al*, 2015; Stoeckius *et al*, 2017; Jackson *et al*, 2020). In contrast, mass spectrometry (MS)‐based proteomics is unbiased in the sense that it measures all proteins within its range of detection (Larance & Lamond, 2015; Aebersold & Mann, 2016). Thus, it would be highly desirable to apply this technology to single cells if the required sensitivity and robustness could be achieved. Previous approaches that employed chemical multiplexing of peptides have labeled a small number of single cells but combined them with a dominant booster channel for MS analysis (Budnik *et al*, 2018; Tsai *et al*, 2020; Schoof *et al*, 2021), which can hamper signal deconvolution (Brenes *et al*, 2019; Cheung *et al*, 2021). Alternatively, proof of principle has been demonstrated for unlabeled approaches using sophisticated sample preparation methods in pico‐liter devices (Li *et al*, 2018; Liang *et al*, 2020; Williams *et al*, 2020).

Here, we set out to develop an ultrasensitive MS‐based workflow that would allow quantitatively precise and accurate MS proteomics data by injecting single cells one by one into the MS—which we call true single‐cell–derived proteomics (T‐SCP). To achieve scale, robustness, and community adoption, we aimed to combine technologies that could readily be made commercially available. We apply our T‐SCP technology to a drug perturbation experiment, capturing functional, dynamic responses on a single‐cell population.

## Results

### Noise‐reduced quantitative mass spectra

**Figure 1:**
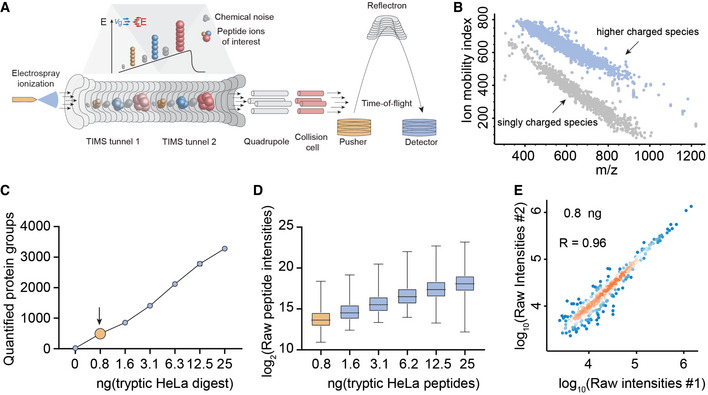
TIMS enables virtually noise‐free spectra and ultra‐high sensitivity proteomics A, BThe TIMS‐qTOF principle separating singly charged background peaks from multiply charged peptide precursor ions, making precursor ions visible at extremely low signal levels (0.8 ng HeLa digest).CQuantified proteins from a HeLa digest dilution series from 25 ng peptide material down to 0.8 ng (arrow), roughly corresponding to the protein amount contained in three HeLa cells on our initial LC–MS setup (See Material and Methods).DLinear quantitative response curve of the HeLa digest experiment in C (Box and Whiskers; The middle represents the median, the top and the bottom of the box represent the upper and lower quartile values of the data, and the whiskers represent the maximum and minimum value of the data).EQuantitative reproducibility of two successive HeLa digest experiments at the lowest dilution (technical LC–MS/MS replicates).

We recently introduced parallel accumulation–serial fragmentation (PASEF), a mass spectrometric acquisition scheme in which peptide ions are released from a trapped ion mobility (TIMS) device into the vacuum system in concentrated packages (Meier *et al*, 2015, 2018). Chemical noise is widely distributed as a result of its heterogeneous nature and the 10‐fold increased peak capacity due to TIMS (Fig 1A and B; Meier *et al*, 2020b). These precursors can be fragmented in a highly sensitive manner, either in data‐dependent (ddaPASEF) or data‐independent (diaPASEF) mode, resulting in very high ion utilization and data completeness (Meier *et al*, 2020a). To explore sensitivity limits of our initial LC‐MS setup (See Material and Methods), we measured a dilution series of HeLa cell lysate from 25 ng down to the equivalent of a few single cells on a quadrupole time‐of‐flight instrument (TIMS‐qTOF). This identified more than 550 proteins from 0.8 ng HeLa lysate with the DDA acquisition mode and a conservative MaxQuant analysis (Fig 1C; Cox & Mann, 2008). Proteins were quantified with the linear signal response expected from the dilution factors (Fig 1D). Furthermore, quantitative reproducibility in replicates at the lowest level was still excellent (R = 0.96, Fig 1E). Given that the protein amount of a single HeLa cell is as low as 150 pg (Volpe & Eremenko‐Volpe, 1970), and accounting for inevitable losses in sample preparation including protein digestion, we estimated that we would need to increase sensitivity by at least an order of magnitude to enable true single‐cell proteomics.

### True single‐cell proteome analysis

**Figure 2:**
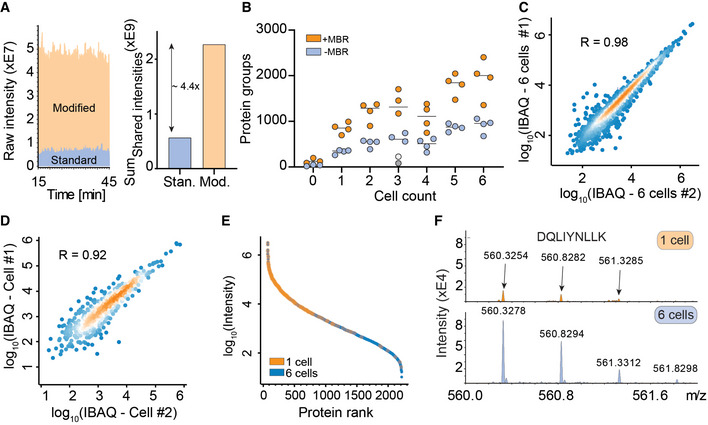
A novel mass spectrometer allows the analysis of true single‐cell proteomes Raw signal increase from standard versus modified TIMS‐qTOF instrument (left) and at the evidence level (quantified peptide features in MaxQuant) (right).Proteins quantified from one to six single HeLa cells, either with “matching between runs” (MBR) in MaxQuant (orange) or without matching between runs (blue). The outlier in the three‐cell measurement in grey (no MBR) or white (with MBR) is likely due to failure of FACS sorting as it identified a similar number of proteins as blank runs (Horizontal lines within each respective cell count indicate median values).Quantitative reproducibility in a rank order plot of a six‐cell replicate experiment.Same as C for two independent single cells.Rank order of protein signals in the six‐cell experiment (blue) with proteins quantified in a single cell colored in orange.Raw MS1‐level spectrum of one precursor isotope pattern of the indicated sequence and shared between the single‐cell (top) and six‐cell experiments (bottom).

**Figure EV1:**
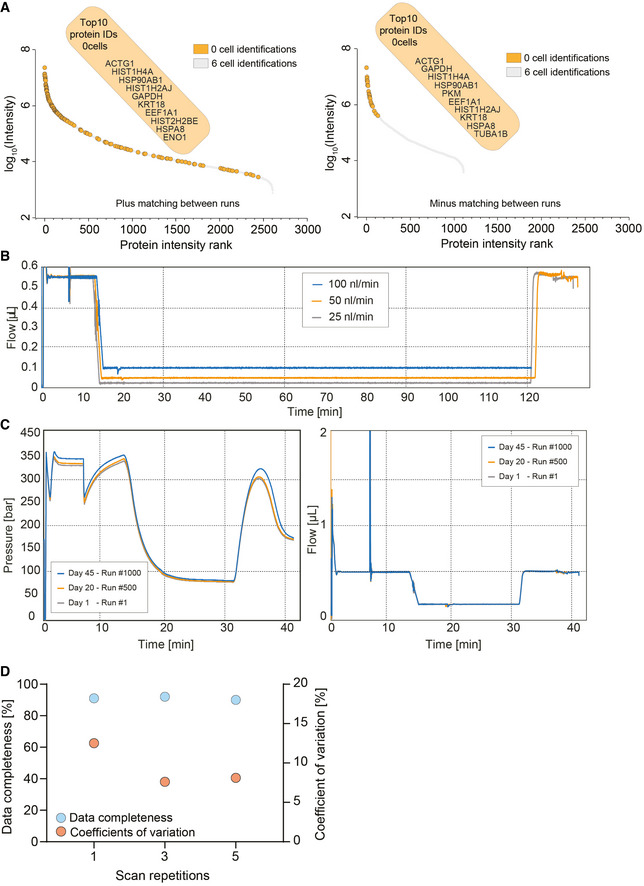
Technical feasibility assessment of ultra‐high sensitivity mass spectrometry and liquid chromatography Ranked protein identifications for six‐cell measurements with and without matching between runs. Zero‐cell protein identifications are highlighted in orange and overlaid on the six‐cell protein rank plot. The top 10 protein identifications of the zero‐cell runs are depicted.True nanoflow at 25, 50, and 100 nl/min flow rate on the EvoSep One liquid chromatography system.Standardized 100 nl/min true nanoflow gradient on the EvoSep One liquid chromatography system. Pressure (Left) and flow profile (right) of the gradient of more than 1,000 consecutive runs (Day 1–Run #1 = gray; Day 20–Run #500 = orange; and Day 45–Run #1,000 = blue).Data completeness (Blue) and coefficient of variation (Orange) evaluation of different diaPASEF consecutive scan repetitions merged for the analysis of 1 ng tryptic HeLa digest. Scans were varied from one, three, and five repetitions.

Three main factors govern MS sensitivity: ionization efficiency, transfer efficiency into the vacuum system, and ion utilization by the instrument (Wilm & Mann, 1996). We first constructed an instrument with a brighter ion source, introduced different ion optic elements and optimized parameters such as detector voltage. Together, this led to a more than fourfold higher ion current (Fig 2A). Next, we FACS sorted zero, one, and up to six single HeLa cells in quadruplicate into individual 384 wells, processed them separately, and analyzed them on this modified mass spectrometer. This resulted on average in 843, 1,279, and 1,890 identified proteins for one, two, and six cells, respectively. Note that this analysis benefited from transferring peptide identifications on the MS1 level, as expected from extremely low sample amounts (Fig 2B). Protein identifications at zero cells are most likely a result of minimal contribution from previous runs since they map to the most abundant proteins of the six‐cell measurements in a rank plot (Fig EV1A and B). Quantitative precision and accuracy were high when comparing single cells, not much reduced from comparing six cells (Fig 2C and D). A rank order abundance plot revealed that the measured single‐cell proteome preferentially mapped to the higher abundant part of the six‐cell proteome, indicating that proteome coverage depended deterministically on overall LC‐MS sensitivity (Fig 2E). Inspecting shared peptides between the single‐cell and six‐cell experiment showed that clearly interpretable precursor isotope patterns were still present at high signal‐to‐noise levels even at single‐cell level following the cell count intensity ratio trend (Fig 2F).

### Ten‐fold sensitivity increase

As electrospray (ES) is concentration dependent, sensitivity increases with decreasing flow rate; however, very low flow systems are challenging to operate robustly and are consequently not widely available (Emmett & Caprioli, 1994; Wilm & Mann, 1996; Greguš *et al*, 2020). We recently described a chromatography system that decouples sample loading and gradient formation from the LC‐MS run and operates at a standardized flow rate of 1 µl/min for high reproducibility (Bache *et al*, 2018). This flow is fully controlled by a single pump instead of the binary gradients produced by other systems. We found that it worked robustly at flow rates down to 25 nl/min but standardized on 100 nl/min, which enabled stable operation for the entire project with the same column‐emitter setup (Fig EV1B and C). ES sprayer diameter and gradient length were optimized for turnover, minimizing carryover and stability.

**Figure 3:**
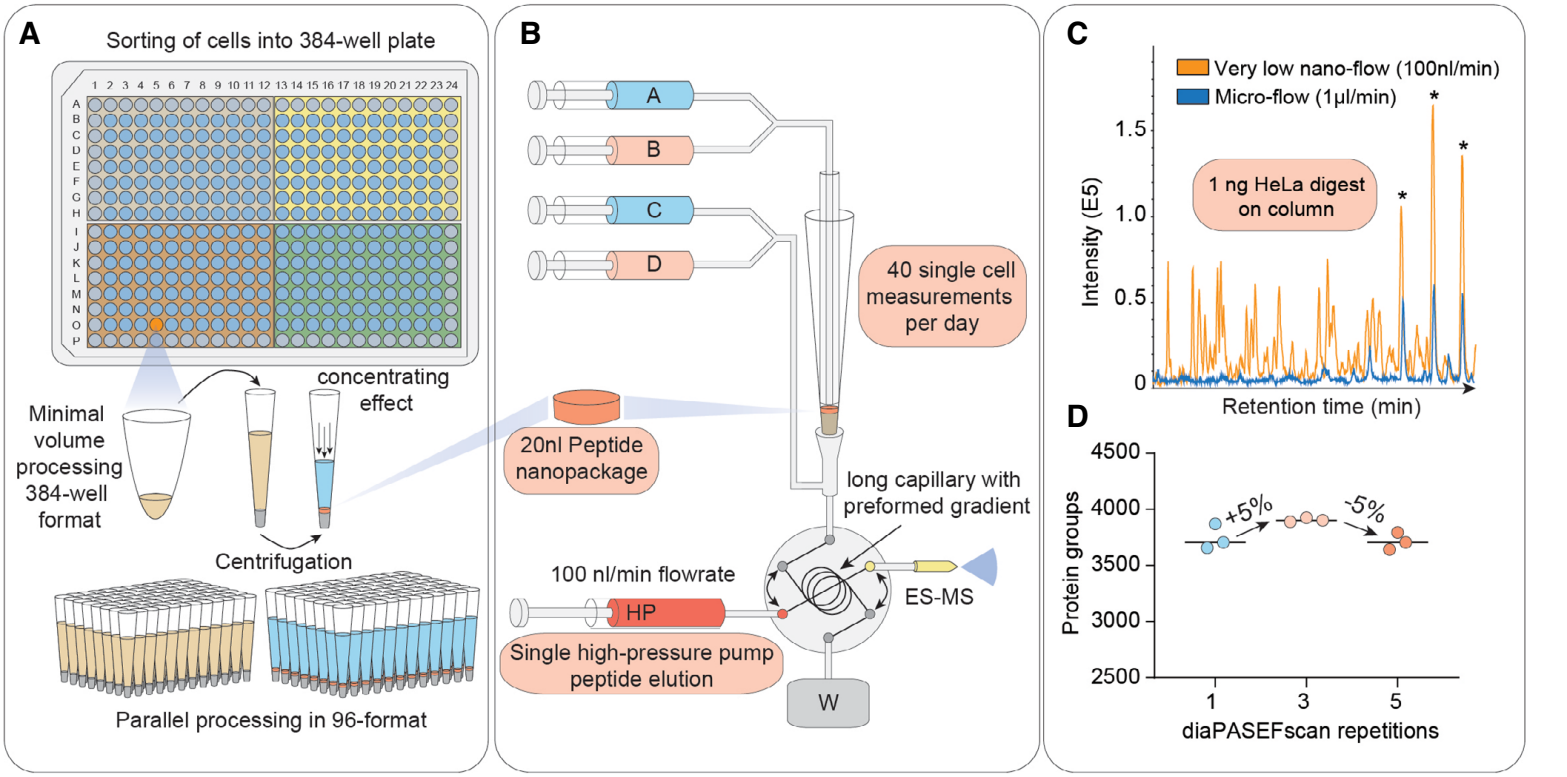
Miniaturized sample preparation coupled to very low‐flow chromatography and diaPASEF Single cells are sorted in a 384‐well format into 1 µl lysis buffer by FACS with outer wells serving as qualitative and quantitative controls. Single cells are lysed and proteins are solubilized at 72°C in 20% acetonitrile, and digested at 37°C. Peptides are concentrated into 20 nl nanopackages in StageTips in a 96‐well format.These tips are automatically picked and peptide nanopackages are eluted in a sub‐100‐nl volume. After valve switching, the peptide nanopackage is pushed on the analytical column and separated, fully controlled by the single high‐pressure pump at 100 nl/min.Base–peak chromatogram of the standardized nanoflow (100 nl/min, orange) and microflow (1 µl/min, blue) gradients with 1 ng of HeLa digest on the StageTip. Asterices indicate polyethylene glycole contaminants in both runs.Nanoflow (100 nl/min) and short‐gradient diaPASEF method combined. Summation of one to five diaPASEF scan repetitions was used to find the optimum for high‐sensitivity measurements at 1 ng of HeLa digest.

MS‐based T‐SCP requires loss‐less sample preparation by protein isolation and solubilization, followed by tryptic protein digestion and peptide purification ready for MS analysis (Budnik *et al*, 2018; Li *et al*, 2018; Zhu *et al*, 2018; Greguš *et al*, 2020; Williams *et al*, 2020). We found that small volumes of weak organic solvents in conical 384‐well plates provided a versatile and automatable environment for efficient cell lysis and protein digestion in minimal volumes (Fig 3A). Briefly, single cells were sorted into wells containing 1 µl lysis buffer, followed by a heating step and further addition of buffer containing digestion enzymes to a total of 2 µl, all in an enclosed space. Peptides were concentrated in a standard EvoTip device, which resembles the functionality of a StageTip (Rappsilber *et al*, 2007)^,^ into 20 nl nanopackages, from which they were eluted in minimal volumes (Fig 3B). To benchmark the effect of reduced flow rate and the concentrated peptide nanopackage elution, we directly compared signal traces of the normal 1 µl/min to the 100 nl/min setup. For 1 ng peptide material, this resulted in a 10‐fold increase in signal (Fig 3C). To achieve high data completeness between hundreds of single‐cell measurements, we next replaced ddaPASEF by diaPASEF, in which fragment‐level matching is further supported by ion mobility data (Meier *et al*, 2020a). We found that combining subsequent diaPASEF scan repetitions further improved protein identification numbers. Together, the very low flow chromatography and this diaPASEF acquisition mode resulted in the highly reproducible identification and quantification of more than 3,900 HeLa proteins from only 1 ng (Fig 3D), a drastic increase from the 550 identified in our initial setup from a similar amount. Data completeness was at 92% and coefficient of variation (CV) < 10% for the selected scan repetition mode (Fig EV1D). This demonstrates that diaPASEF provides its advantages also at extremely low sample amounts, prompting us to adopt this acquisition mode for the single‐cell workflow in the remainder of this work.

### T‐SCP dissects arrested cell cycle states

**Figure 4:**
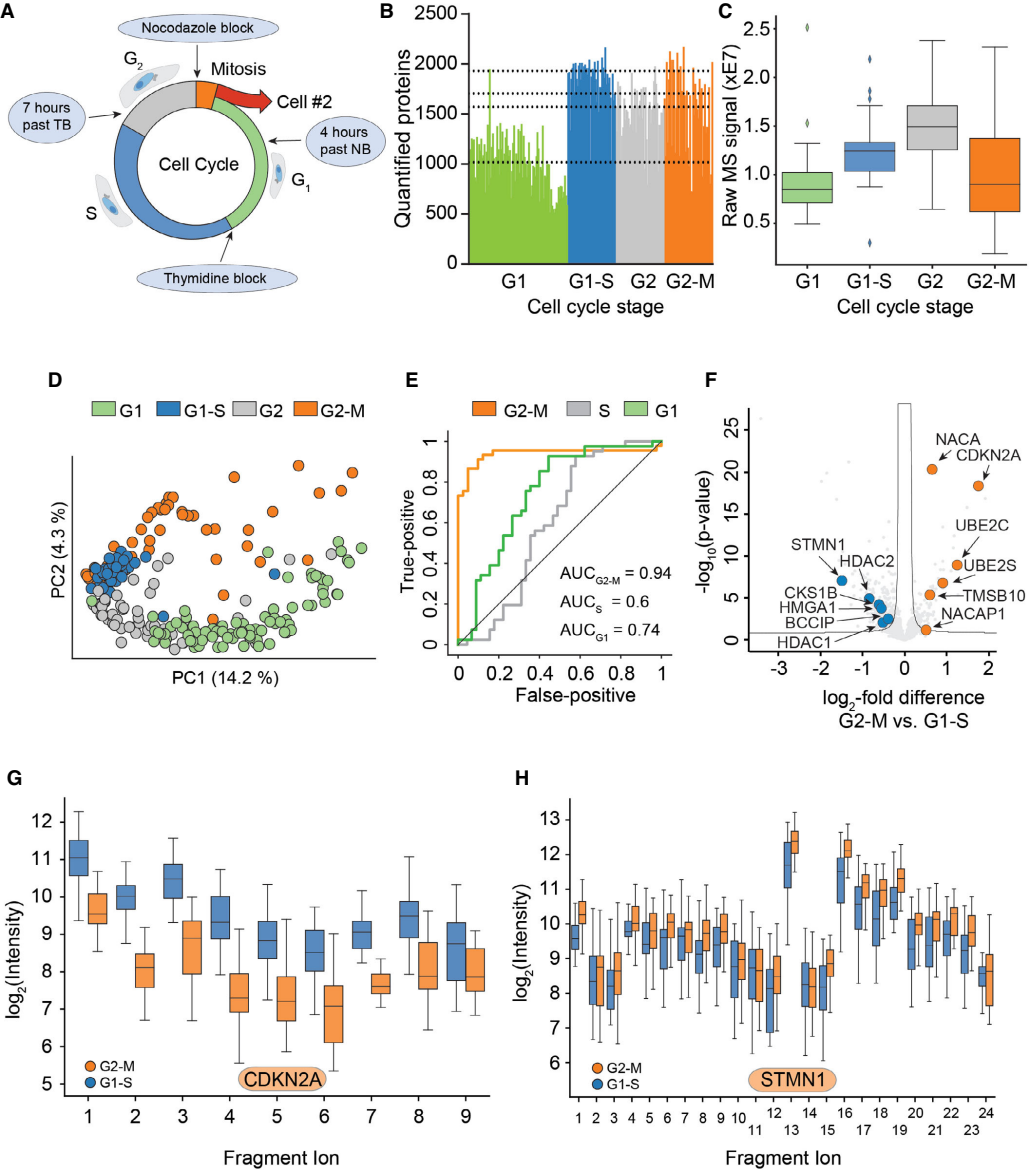
T‐SCP correctly quantifies cell cycle states Arresting single cells by drug perturbation.Numbers of protein identifications across 231 cells in the indicated cell cycle stages as enriched by the drug treatments in A (Dashed lines indicate the median number of identifications for each respective cell cycle stage).Boxplot of total protein signals of the single cells in B after filtering for at least 600 protein identifications per cell and 15% data completeness per protein across cells (G1: *n* = 84; G1‐S: *n* = 41; G2: *n* = 52; and G2‐M: *n* = 45); (Box and Whiskers; The middle represents the median, the top and the bottom of the box represent the upper and lower quartile values of the data, and the whiskers represent the 1.5× IQR).PCA of single‐cell proteomes of B.Receiver operator curves (ROC) for the distinction between G2‐M cells and G1‐S cells based on sets of marker proteins for G1, S, and G2‐M phase, respectively, with the indicated area under the curve (AUC) scores. G1‐S cells were used as positive targets for the G1 and S score, G2‐M for the G2‐M score.Volcano plot of quantitative protein differences in the two drug‐arrested states. Arrows point toward colored significantly regulated key proteins of interest (Benjamini–Hochberg corrected multiple‐sample *t*‐test; FDR = 0.05; S = 0.2).Quantitative fragment ion‐level data of CDKN2A‐associated peptides (FDR < 10^−15^; Benjamini–Hochberg corrected multiple‐sample *t*‐test (Box and Whiskers; The middle represents the median, the top and the bottom of the box represent the upper and lower quartile values of the data, and the whiskers represent the 1.5× IQR).Quantitative fragment ion‐level data of STMN1‐associated peptides (FDR < 10^−15^; Benjamini–Hochberg corrected multiple‐sample *t*‐test (Box and Whiskers; The middle represents the median, the top and the bottom of the box represent the upper and lower quartile values of the data, and the whiskers represent the 1.5× IQR).

**Figure EV2:**
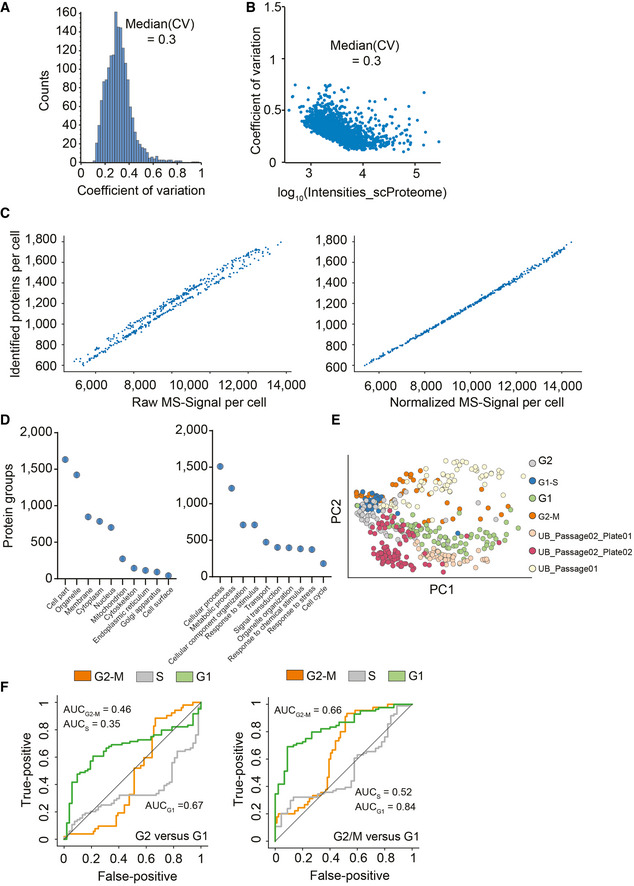
True single‐cell proteomics data set description Frequency plot for coefficient of variation occurrence within the 420 single‐cell proteomics data set.Protein log_10_ intensity versus coefficient of variation.Raw log(x + 1)‐transformed intensity values of proteins per cell plotted against the number of identified proteins per cell (Left) and after normalization by local regression to cancel out those differences to enable downstream analysis (Right).Principal component analysis of cell cycle stage enriched single‐cell proteomics measurements and three cell culture batches projected on top.Category count of gene ontology annotations for cellular compartment and biological process terms. Exemplary, category count terms are shown for the cellular compartment (Left) and biological process (Right) for more than 430 single‐cell proteomics data set.Cell cycle stage prediction for G2 versus G1 phase cells (Left) and G2/M versus G1 phase cells (Right) using the 60 topmost differentially expressed proteins reported by Geiger and coworkers (Aviner *et al*, 2015) as input.

The cell cycle is an important and well‐studied biological process that has frequently been used as a test case in single‐cell studies (Aviner *et al*, 2015; Ly *et al*, 2017). To investigate if our proteomics workflow could detect biological responses to drug perturbation at the single‐cell level, we treated HeLa cells with thymidine and nocodazole to produce four cell populations enriched in specific cell cycle stages (231 cells; Fig 4A). We quantified up to 2,083 proteins per single cell and 2,501 overall using a HeLa dia spectral library with about 4,000 protein groups. This number ranged from a median of 1,018 in G1 to 1,932 in G1/S, 1,572 in G2, and 1,705 in G2/M (Fig 4B). The full data set, even though biologically heterogeneous, showed a median coefficient of variation (CV) of 0.3 across all genes and a clear dependence of the CV on protein intensity levels (Fig EV2A and B). To estimate the total protein amount per cell, we summed all protein signals based on their identifying peptides. Judged by protein amount, G2 cells were approximately 1.8‐fold larger than G1 cells; thus, T‐SCP correctly reflected the proliferation state, while highlighting a substantial heterogeneity within each cell cycle stage that would have been hidden in bulk sample analysis (Fig 4C). To be able to directly compare single‐cell proteomes and cancel out protein abundance differences attributed to varying total protein amounts and identifications of each cell, we normalized our data using the retention time‐dependent normalization module offered by the search engine DIA‐NN across cells (Demichev *et al*, 2020; Fig EV2C). Furthermore, we stringently filtered our data set for at least 600 protein identifications per cell and more than 15% observations for each protein across remaining single cells (See Material and Methods). The proteomes of the different cell cycle states grouped together in a principal component analysis (PCA) plot (Fig 4D). In addition to these drug‐perturbed cells, we measured more than 200 untreated ones from two independent cell culture batches. The proteomes of these asynchronous cells distributed well across the cell cycle states, while different passage batches were enriched in the G1 and G2 phase (Fig EV2D). This highlights that biological variation dominates remaining technical variation. The T‐SCP data set covered proteins assigned to many cellular compartments, membranes, and biological processes involved in biological regulation, metabolism, transport, and signal transduction at high quantitative precision despite severe systematic perturbation introducing stark biological variation and proteome remodeling (Fig EV2E, Dataset EV1).

Next, we asked whether single‐cell proteome measurements can be used to assign cellular states, similar to how single‐cell RNA sequencing (scRNA‐seq) measurements have frequently been applied to cell type and state discovery, highlighted by cellular atlas projects (Regev *et al*, 2017). In previous proteomics studies, cell populations had been enriched for cell cycle states and sets of regulated proteins had been extracted (Aviner *et al*, 2015; Ly *et al*, 2017). We here selected cell cycle stage marker proteins as the top 60 most differentially expressed in the G2/M‐, G1‐, or S‐phase protein set from Geiger and coworkers (Aviner *et al*, 2015), as it used similar drug treatment on bulk populations and investigated how likely cells from different cell cycle stages could be distinguished (Dataset EV2). We used these marker proteins to set up cell cycle stage‐specific scores indicating the likelihood to belong to the respective phase previously used for scRNA‐seq cell cycle stage predictions. This model clearly distinguished cells from G2/M and G1/S and also other comparisons (Figs 4E and EV2F; Wolf *et al*, 2018).

**Figure EV3:**
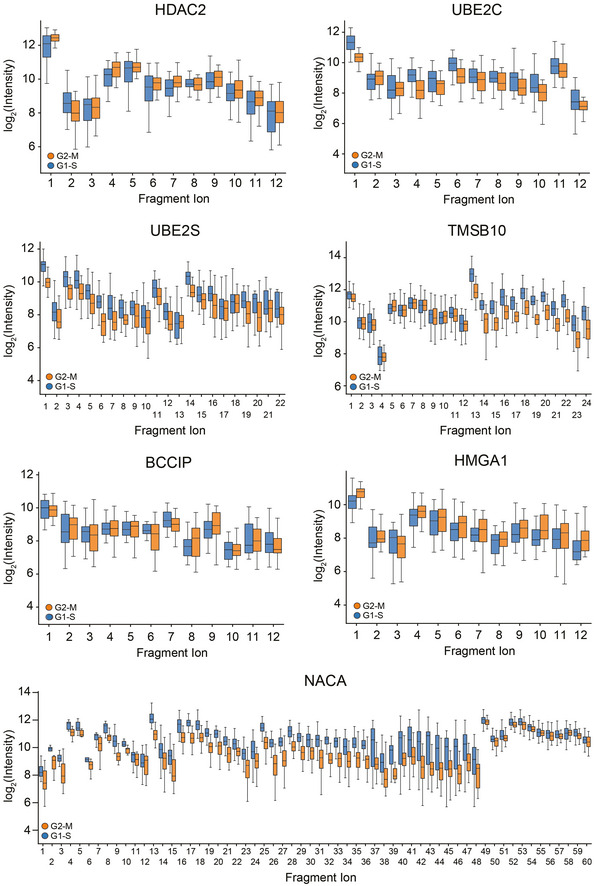
Peptide fragment ion intensities of several proteins Fragment ion intensities of peptides for several differentially expressed proteins (HDAC2, FDR = 1.2E‐3; UBE2C, FDR = 4.7E‐6; UBE2S, FDR = 4.1E‐15; TMSB10, FDR = 4.1E‐15; BCCIP, FDR = 4.7E‐1; HMGA1, FDR = 1.4E‐2; NACA, and FDR = 4.1E‐15) in the comparison of nocodazole‐ (G2‐M transition) and thymidine (G1‐S transition)‐treated cells. Boxplots represent the intensity distribution of indicated peptide fragment ion intensities.

Next, we investigated the differentially expressed proteins between the drug‐arrested cell cycle stage transition G2/M and G1/S. Among the significantly regulated proteins was a large number of known cell cycle regulators, some of which are highlighted (Dataset EV3; Fig 4F). Quantitative MS data at the fragment ion level were highly significant for these as illustrated by the cell cycle regulator CDKN2A, STMN1, and further examples (FDR < 10^−15^, Figs 4G and H, and EV3). Our single‐cell data set also highlighted proteins not previously associated with the cell cycle and the G2/M transition. For instance, NACA was clearly identified and regulated (FDR < 10^−15^, Fig EV3).

### SC proteomes compared to transcriptomes

Given our set of more than 430 single‐cell proteomes, we compared the T‐SCP measurements after filtering with similar single‐cell RNA sequencing data (scRNA‐seq) (Hu *et al*, 2019; Schwabe *et al*, 2020). To achieve technology‐independent insights, we selected assays from two widespread scRNA‐seq technologies, Drop‐seq (Macosko *et al*, 2015) and the lower‐throughput SMART‐Seq2 (Picelli *et al*, 2014), on the same cellular system. The Drop‐seq assay is based on unique molecular identifiers (UMIs) to control for amplification biases in library preparation, whereas the SMART‐Seq2 assay is not UMI controlled. Note that MS‐based proteomics inherently does not involve any amplification and is not subject to associated artifacts.

**Figure EV4:**
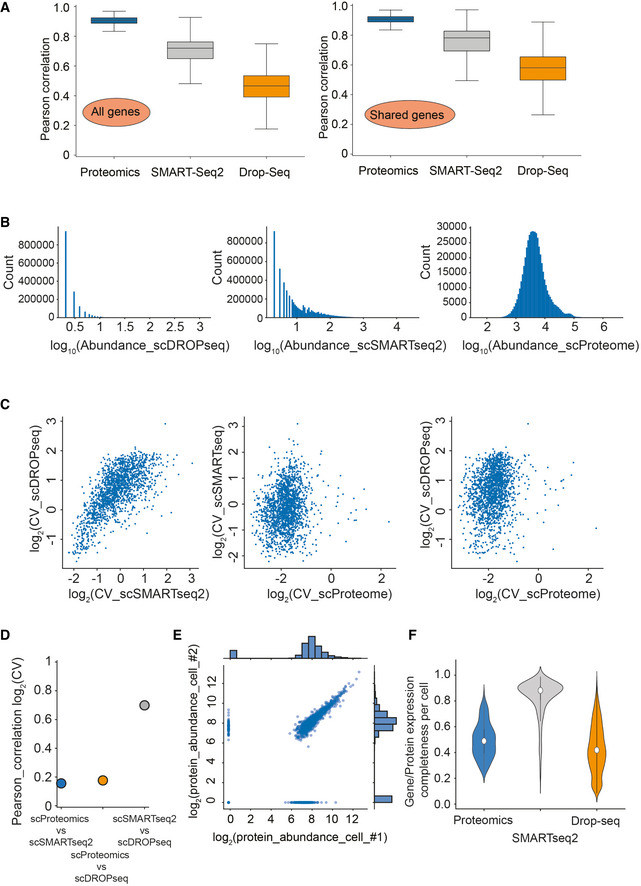
Correlation and gene/protein completeness analysis of single‐cell transcriptome sequencing and our LC–MS‐based single‐cell proteomics data set of the same cell line Pearson correlation of observations for each cell within each of the technologies on all genes (MS‐based proteomics, SMART‐Seq2 RNA sequencing, and droplet‐based RNA sequencing; Left) and for each cell within each of the technologies on shared genes between technologies (MS‐based proteomics, SMART‐Seq2 RNA sequencing, and droplet‐based RNA sequencing; Right).Gene/Protein expression completeness per cell on all shared genes between the three technologies (scProteomics; SMART‐seq2; and DROP‐seq).Gene and protein expression completeness as a function of ranked genes/proteins for all three technologies (Proteomics, DROP‐seq, and SMART‐Seq2). Arrows indicate a bimodal distribution for single‐cell RNAseq data in both technologies, which is absent in proteomics.Data completeness across single cells as a function of mean protein abundance for MS‐based single‐cell proteomics and both single‐cell RNA sequencing (Drop‐Seq, SMART‐Seq2). Expected poison dropout distribution shown in red.Scatter plot of two independently measured single‐cell proteome expression values.

**Figure 5:**
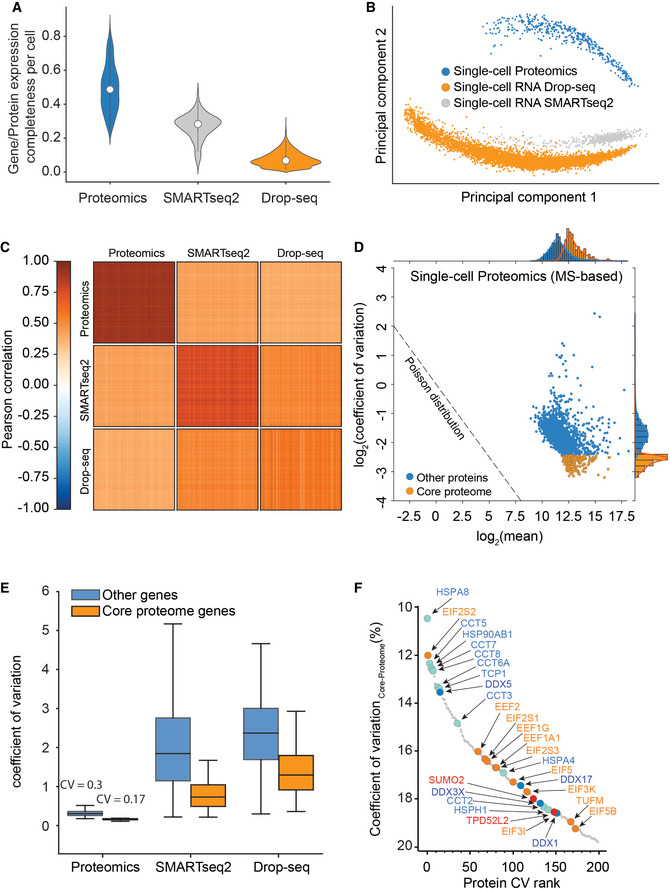
Single cells have a stable‐core proteome but not transcriptome Gene or protein expression completeness per cell for T‐SCP (Cells × Proteins: 424 × 2,480), SMARTseq2 (Cells × Genes: 720 × 24,990), or Drop‐seq (Cells × Genes: 5,022 × 41,161) shown as violin plot; middle points represent the data set median.Principal component analysis of single‐cell gene and protein expression measurements (1,672 shared genes).Heat map of cell–cell correlations across individual cells measured by proteomics and by both transcriptome technologies (1,672 shared genes).Coefficient of variation of single‐cell protein expression levels in LC‐MS based proteomics as a function of mean expression levels with the “core proteome” colored in orange.Boxplot of coefficient of variation of protein and transcript expression levels in LC‐MS based proteomics, SMARTseq2, and Drop‐seq technologies with a separate “core proteome” colored in orange (Box and Whiskers; The middle represents the median, the top and the bottom of the box represent the upper and lower quartile values of the data, and the whiskers represent the 1.5× IQR).Rank order abundance plot for the core proteome with color‐coded protein classes (Red: SUMO2 and TDP52L2 proteins; Turquoise: Chaperonin and folding machinery‐associated proteins. Orange: Translation initiation and elongation; Yellow: Structural proteins; Blue: DEAD box helicase family members).

Despite subtle differences, HeLa cell culture should reflect a characteristic global distribution of gene and protein expression states (Liu *et al*, 2019). This assumption would allow us to assess self‐consistency of the measurement technologies. First, we computed the distribution over all pairwise correlation coefficients of cells within a technology (Svensson, 2020). We found that in the proteome measurement, cells have higher correlation on average than in the droplet‐based and the SMART‐Seq2 method (Fig EV4A). This is true when analyzing all available genes within each particular data set and also when analyzing all shared genes (Fig EV4A). Comparing all three data sets for gene or protein expression completeness (on average in 49% of the 2,480 proteins observed by MS‐based proteomics), protein expression completeness per cell followed a normal distribution (Fig 5A). For SMART‐Seq2, this was only 27 and 8% in the droplet‐based protocol. Furthermore, when comparing the gene or protein expression completeness on shared gene level, the saturation sequencing effect in the SMARTseq2 data set becomes pronounced. In the Drop‐seq data set, this is controlled for using UMI‐based protocols (Islam *et al*, 2014; Svensson, 2020; Fig EV4B). Both single‐cell RNA‐sequencing technology data sets followed a bimodal gene completeness frequency distribution, while single‐cell proteomes do not (Fig EV4C).

Next, we investigated whether there were systemic limitations of the detection in the protein measurements. Such effects are discussed for scRNA‐seq measurements as “drop‐out events” or “zero‐inflation,” although they are now much reduced in UMI‐based protocols (Islam *et al*, 2014; Svensson, 2020). We identified signs of such detection limits as bimodality in the lower abundance range of the protein measurements (Fig EV4C–E). This suggests that—apart from increased sensitivity—our single‐cell protein analysis could benefit from imputation or tailored likelihood‐based parameter estimation methods (Risso *et al*, 2018; Lähnemann *et al*, 2020).

For bulk measurements, transcript levels generally correlate moderately with the corresponding protein levels, however, this correlation strongly depends on the biological situation (Buccitelli & Selbach, 2020). At the single‐cell level, this effect is further convoluted by dissimilar measurement technologies and possibly by fundamental biological differences between the transcriptome and proteome. We asked to what degree scRNA‐seq measurements could be used as a proxy for protein measurements in our data but found that protein measurements separate strongly from RNA in a principal component analysis (Fig 5B). While single‐cell transcript expression levels correlate well between the scRNA‐seq technologies, they diverge from single‐cell protein measurements (Fig 5C).

**Figure EV5:**
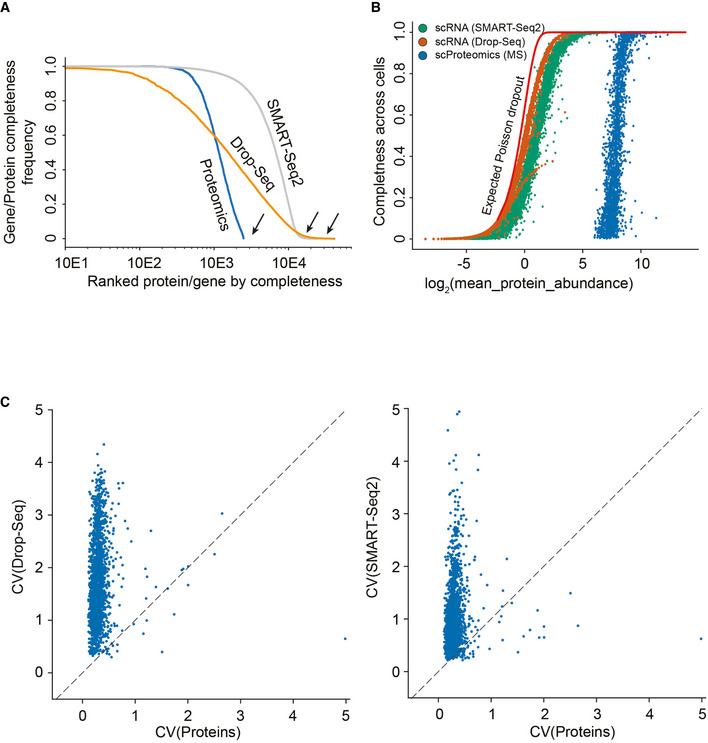
Data distribution analysis of single‐cell transcriptome sequencing and our LC–MS‐based single‐cell proteomics data set of the same cell line Histogram of log_10_ abundance of scDROPseq (left), scSMARTseq2 (middle), and scProteomics data (right).The coefficient of variation of a gene measured by either Drop‐Seq technology (Left) or SMART‐Seq2 (Right) compared to the coefficient of variation of the corresponding protein measured by MS‐based single‐cell proteomics.Pearson correlation of coefficients of variation for each gene shared within each comparison.

As the transcriptome is measured as count data and the proteome as signal intensity levels, we further investigated the correlation between the transcriptome and proteome by correlating the coefficients of variation for shared genes between all data sets (Fig EV5A). This analysis reflects the quantitative variation of each gene at the single‐cell transcriptome and single‐cell proteome level across the cell cycle. Indeed, we found that the quantitative variation for the single‐cell transcriptomes was high and correlation was consistent between both data sets (Fig EV5B).

In stark contrast, gene‐level variation of both technologies did not correlate well with the single‐cell proteome, highlighting again that both biological information levels are regulated fundamentally different at the single‐cell level (Fig EV5C). This suggests that single‐cell protein and RNA levels are very different, re‐emphasizing that protein measurements yield complementary information to RNA measurements and do not simply re‐iterate similar gene expression states. This implies distinct RNA and protein abundance regulation mechanisms on both modalities, dissection of which would not be possible with RNA measurements alone.

### T‐SCP reveals a stable core proteome

**Figure EV6:**
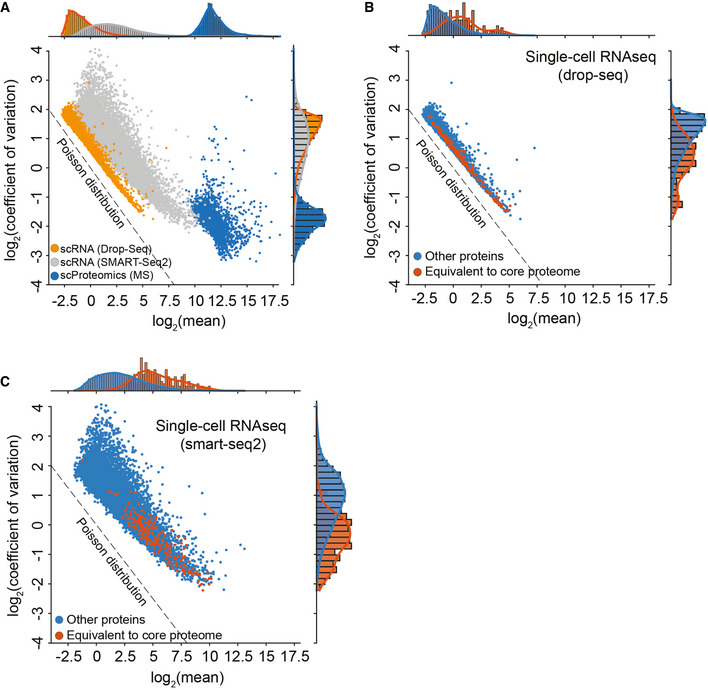
Single‐cell core proteome distribution compared to matched gene expression distribution in single‐cell transcriptome data of the same cell line Coefficient of variation distribution as a function of log_2_ mean gene or protein intensities for Drop‐Seq (Orange), SMART‐Seq2 (Gray), or MS‐based single‐cell proteomics (Blue). Expected Poisson distribution shown as dashed line.Coefficient of variation of single‐cell RNA‐sequencing (drop‐seq) levels as a function of mean expression levels with the “core proteome” colored in orange and non‐“core proteome” genes in blue. Expected Poisson distribution shown as dashed line.Coefficient of variation of single‐cell RNA‐sequencing (smart‐seq2) levels as a function of mean expression levels with the “core proteome” colored in orange and non‐“core proteome” genes in blue. Expected Poisson distribution shown as dashed line.

Prompted by the divergent correlation values between the proteome and transcript levels, we next investigated the variability of gene expression as a function of abundance. For protein expression measurements, coefficients of variation were very small across covered abundances (Figs 5E and EV5A). This is consistent with a model in which the covered proteome is stable and probed deterministically across its full dynamic range. In contrast, the same analysis for UMI‐controlled and not UMI‐controlled scRNAseq data revealed a much higher overall transcriptome variability, as measured by the coefficient of variation of single‐cell RNA‐seq compared to protein measurements (Figs 5D and EV6A). Remarkably, this difference is already very apparent with the current sensitivity of MS‐based proteomics, which will surely increase in the future. Comparing single‐cell proteome measurements with six‐cell proteomes (Fig 2C) suggests that a moderate increase in MS sensitivity would reveal a large part of the proteome to be quantitatively stably expressed.

We also observed that the single‐cell transcriptome is dominated by shot noise, which has a Poisson distribution, because many of the transcripts are expressed at lower than one copy per cell on average (Fig EV6A). This means that a given single cell can have zero, one, or two transcripts of many of its expressed genes, leading to a Poisson distribution when summing up many single‐cell measurements. For genes with higher expression values, there will always be transcripts present in each single cell and then the expression distribution of those genes is not shot noise dominated. For proteins, in contrast, the CVs depend only on the measurement sensitivity, as there are always sufficient copies in each cell to ensure that their expression levels are not biologically shot noise limited (Fig EV6A and 2A).

Based on these observations, we defined a “core‐proteome” subset in the MS‐based proteomics data by selecting the top 200 proteins with the lowest CVs of the proteins shared between at least 70% of the more than 430 single cells, including the drug perturbations (Dataset EV4). Interestingly, these proteins were distributed well across the covered dynamic range of the proteome (Fig 5D). Strikingly, we found the corresponding transcripts of the core proteome to be distributed across the full range of CVs in single‐cell transcriptome data (Figs 5E and EV6B and C). The core proteome highlighted proteins frequently used for normalization such as HSP90, providing a positive control (Fig 5F). The CV rank plot of the core proteome also reveals a diverse set of proteins, including representatives of translation initiation and elongation, folding machineries, and nucleic acid helicases. Interestingly, we also identify TPD52L2 as one of the most stable proteins, which in turn is described as one of the most abundant proteins in HeLa cells (Hein *et al*, 2015) and SUMO2, which is known for its involvement in a plethora of essential regulatory cellular processes, suggesting a stable cellular SUMO2 pool even during stark proteome remodeling (Gareau & Lima, 2010).

## Discussion

The T‐SCP pipeline combines miniaturized sample preparation coupled to very low‐flow liquid chromatography and a novel mass spectrometer resulting in at least one order of magnitude sensitivity gain at highest robustness for the analysis of single cells. We quantify cellular heterogeneity following targeted perturbation, which enables the direct analyses of drug responses in single‐cell hierarchies on the proteome level. Furthermore, the comparison of single‐cell RNA and proteome level revealed that the proteome is stable while the transcriptome is more stochastic, highlighting substantial regulation of translation and setting the stage for its elucidation at the single‐cell level.

Although mainly demonstrated here for single‐cell total proteome measurements, the sensitivity gain achieved in our workflow will be advantageous in any situation that is sample limited. This includes investigation of other compound classes such as metabolites or drugs, post‐translational modifications from small numbers of cells or from *in vivo* material, and measurements directly from paraffin‐embedded formalin‐fixed (FFPE) pathology specimens, which we are already pursuing (preprint: Bhatia *et al*, 2021; preprint: Mund *et al*, 2021).

Our ion mobility‐enhanced workflow is also compatible with chemical multiplexing with the advantage that the booster channel causing reporter ion distortions could be omitted or reduced (Ogata & Ishihama, 2020) and also benefit alternative multiplexing strategies like complementary TMT or EASI‐Tag (Wühr *et al*, 2012; Winter *et al*, 2018). Furthermore, there are many opportunities for increasing overall sensitivity, including even brighter ion sources, improved chromatography, and better data analysis and modeling tools, similar to the rapid recent advances in the scRNAseq field.

## Material and Methods

### Reagents and Tools table

**Table:** 

**Chemicals, enzymes and other reagents**		
Formic acid	Sigma Aldrich/Merck	Cat # 64‐18‐6
Acetonitrile	Sigma Aldrich/Merck	Cat # 75‐05‐8
Trifluoroacetic acid	Sigma Aldrich/Merck	Cat # 76‐05‐1
Water, Optima™ LC/MS Grade	Fisher Chemical	Cat # W64
Lysyl‐Endopeptidase	Wako Chemicals	Cat # 129‐02541
Trypsin	Sigma Aldrich/Merck	Cat # T6576
**Software**		
MaxQuant (1.6.7.0)	https://maxquant.org/	N/A
Perseus (1.6.7.0)	https://maxquant.org/perseus/	N/A
Jupyter Notebook	https://jupyter.org/	N/A
**Other**		
384‐Well Plates	Eppendorf	Cat # 0030129547
Adhesive PCR Sealing Foil Sheets	Thermo Scientific	Cat # AB‐0626
Empore SPE SDB‐RPS disk	Sigma Aldrich/Merck	Cat # 66886‐U
iST' sample preparation kit	PreOmics GmbH	Cat # P.O. 00001
ThermoMixer^®^	Eppendorf	Cat # 460‐0223
NanoDrop™ One/OneC Microvolume UV‐Vis Spectrophotometer	Thermo Fisher	Cat # ND‐ONEC‐W
Concentrator plus	Eppendorf	Cat # F‐45‐48‐11
Mastercycler X50h	Eppendorf	Cat # 63160000
EASY‐nLC™ 1200 System	Thermo Fisher	Cat # LC140
EvoSep One	EvoSep	Cat # EV‐1000
EvoTip	EvoSep	Cat # EV‐2001
15 cm, 75 µm ID, 1.9 µm ID beads with 120A surface	EvoSep	Cat # EV‐1112
ZDV Emitter Sprayer 10 µm ID	Bruker Daltonik GmbH	Cat # 1865691
timsTOF Pro	Bruker Daltonik GmbH	N/A
timsTOF SCP	Bruker Daltonik GmbH	N/A
Column oven	Sonation lab solutions	Cat # PRSO‐V2

### Methods and Protocols

#### Sample preparation for bulk dilution experiments

For all benchmark experiments, purified peptides from bulk HeLa cells were used. HeLa was cultured in Dulbecco’s modified Eagle’s medium at 10% fetal bovine serum, 20 mM glutamine, and 1% penicillin–streptomycin. Cells were collected by centrifugation, washed with phosphate‐buffered saline (PBS), flash frozen in liquid nitrogen, and stored at −80°C. Cells were resuspended in PreOmics lysis buffer (PreOmics GmbH) and boiled for 20 min at 95°C, 1,500 rpm to denature, reduce, and alkylate cysteins, followed by sonication in a Branson, cooled down to room temperature, and diluted 1:1 with 100 mM Tris–HCl pH 8.5. Protein concentration was estimated by nanodrop measurement and 500 µg were further processed for overnight digestion by adding lysC and trypsin in a 1:50 ratio (µg of enzyme to µg of protein) at 37°C and 1,500 rpm. Peptides were acidified by adding 1% trifluoroacetic acid (TFA) and 99% isopropanol (IprOH) in a 1:1 ratio, vortexed, and subjected to StageTip (Rappsilber *et al*, 2007) clean‐up via styrenedivinylbenzene reverse‐phase sulfonate (SDB‐RPS). Twenty microgram of peptides were loaded on two 14‐gauge StageTip plugs. Peptides were washed two times with 200 µl 1% TFA and 99% IprOH followed by 200 µl 1% TFA and 99% IprOH in an in‐house‐made StageTip centrifuge at 2,000 g and elution with 100 µl of 1% Ammonia, 80% acetonitrile (ACN), 19% ddH_2_O into PCR tubes, and finally dried at 60°C in a SpeedVac centrifuge (Eppendorf, Concentrator plus). Peptides were resuspended in 0.1% TFA, 2% ACN, and 97.9% ddH_2_O.

#### Sample preparation for single‐cell experiments (Protocol style)

1. HeLa cells were cultured following a standard protocol as described above.
2. Supernatant was removed, cells were detached with trypsin treatment, followed by strong pipetting for cell aggregate dissociation.
3. Cells were washed three times with 500 µl ice‐cold phosphate‐buffered saline (PBS), pelleted by centrifugation, and the supernatant was removed.
4. For fluorescent‐activated cell sorting (FACS), 5 µl DAPI was added to the 5 ml single‐cell solution and sorting performed on the DAPI‐negative live cell population.
5. Single cells were sorted into 384‐well TwinTec Eppendorf plates containing 1 µl of 20% acetonitrile (ACN), 100 mM Tris–HCl pH 8.5, centrifuged briefly, sealed with aluminum foil and frozen at −80°C until further use (we cannot exclude that FACS sorting could lead to subtle changes in the proteome).
6. Single‐cell containing 384‐well plates were incubated for 30 min at 72°C in a PCR cycler, followed by 5 min sonication (Elmasonic P) at 37 kHz and room temperature.
7. Protein digestion was performed overnight at 37°C in a PCR cycler after adding 1 µl of 20% ACN, 100 mM Tris–HCl, pH 8.5, and 1 ng trypsin/lysC mix. (For the peptide bulk and cell count dilution experiments, peptides were resuspended in 4 µl of 2% ACN, 0.1% TFA, and 97.9% ddH_2_O, and injected directly via NanoLC.)
8. Samples were dried in a SpeedVac at 30°C for 45 min
9. Single‐cell‐derived peptides were resuspended in 5 µl pure formic acid and incubated for 10 min on a thermo shaker at 25°C and 800 rpm.
10. EvoTips were activated following the standard EvoSep protocol (Sample loading protocol for Evotips). Then, 50 µl buffer A was added to each EvoTip followed by centrifugation at 200 g for 1 min (This leaves approximately 30 µl of buffer A on top of the SPE material.)
11. 15 µl of buffer A (99.9% ddH_2_O, 0.1% FA) were added to each single‐cell well containing the dissolved single‐cell peptides in 5 µl FA, followed by a 5‐min shaking phase on a thermoshaker at 800 rpm, RT.
12. The single‐cell peptides (20 µl total now) were transferred into the activated EvoTip, followed by centrifugation at 600 g for 1 min and two centrifugation steps after adding 50 µl buffer A. Last, 150 µl buffer A was added to each EvoTip and spun for 30 s at 300 g.

#### Cell cycle experiments

The drug‐perturbed cell cycle arrest experiment was designed to enrich cells in four cell cycle stages—G1, the G1/S transition, G2, and the G2/M transition. HeLa cells were grown to approximately 30% confluence as described above, washed and treated for 24 h with 5 mM thymidine, released for 4.5 h, and treated again with 5 mM thymidine or 0.1 µg/ml nocodazole for 13 h. Cells of the G1/S phase (thymidine block) or G2/M phase (nocodazole block) were washed in PBS, trypsinated, subjected to strong pipetting to dissociate cell aggregates, and ice‐cold PBS washes before DAPI‐negative single live cell FACS sorting. A second set of G1/S phase and G2/M phase blocked cells was washed and cultured for 7 h or 2.5 h to enrich early G2 and G1 phase HeLa cells. These were washed with PBS, trypsinated, and subjected to DAPI‐negative single live cell FACS sorting into 384‐well plates pre‐loaded with 1 µl 20% acetonitrile, 100 mM Tris–HCl, pH 8.5 lysis buffer. Furthermore, we prepared presumable unsynchronized cells sets from two independent cell cultures and subjected them to sample preparation as described below.

#### High‐pH reversed‐phase fractionation

To generate a deep library of HeLa precursors for all data‐dependent benchmark experiments, peptides were fractionated at pH 10 with the spider fractionator (Kulak *et al*, 2017). Fifty micrograms of purified peptides were separated on a 30 cm C_18_ column in 96 min and concatenated into 24 fractions with 2 min exit valve switches. Peptide fractions were dried in a SpeedVac and reconstituted in 2% ACN, 0.1% TFA, and 97.9% ddH_2_O for LC–MS analysis.

#### Liquid chromatography

For the initial benchmark experiments with HeLa bulk dilution and the cell count dilution, liquid chromatography analysis was performed with an EASY nanoLC 1200 (Thermo Fisher Scientific). Peptides were loaded on a 45 cm in‐house packed HPLC column (75 µm inner diameter packed with 1.9 µm ReproSil‐Pur C18‐AQ silica beads, Dr. Maisch GmbH, Germany). Sample analytes were separated using a linear 60 min gradient from 5 to 30% B in 47.5 min followed by an increase to 60% for 2.5 min, by a 5 min wash at 95% buffer B at 300 nl/min, and re‐equilibration for 5 min at 5% buffer B (buffer A: 0.1% formic acid (FA) and 99.9% ddH_2_O; buffer B: 0.1% FA, 80% ACN, and 19.9% ddH_2_O). The column temperature was kept at 60°C by an in‐house manufactured oven.

For all other proteome analyses, we used an EvoSep One liquid chromatography system (Bache *et al*, 2018) and analyzed the single‐cell proteomes with a novel 35 min stepped pre‐formed beta gradient eluting the peptides at 100 nl/min flow rate. We used a 15 cm × 75 μm ID column with 1.9 μm C18 beads (EvoSep) and a 10 µm ID zero dead volume electrospray emitter (Bruker Daltonik). Mobile phases A and B were 0.1% FA in water and 0.1% FA in ACN, respectively.

Both LC systems were coupled online to a modified trapped ion mobility spectrometry quadrupole time‐of‐flight mass spectrometer (timsTOF Pro, Bruker Daltonik GmbH, Germany) via a nanoelectrospray ion source (Captive spray, Bruker Daltonik GmbH).

#### Construction of a novel mass spectrometer with higher sensitivity

We updated our ion source to draw more ions into the vacuum system of the instrument. This is accomplished by modifying the glass capillary that conducts gas and ions between the ionization region at atmospheric pressure and the first pumping region. The added gas is eliminated by an additional pumping stage and associated prototype ion optics. These ion optics—a high pressure ion funnel and a RF multipole—confine the ions while the added gas is removed and moves them to the next vacuum region where TIMS analysis occurs. Importantly, the glass capillary is oriented orthogonal to the high‐pressure funnel (as in prior designs) so that neutral contaminants and solvent droplets are directed by the gas flow away from the funnel. Furthermore, the high‐pressure funnel and RF multipole are oriented orthogonal to the TIMS. This has the dual advantage of maintaining the gas dynamics of our original design, which is crucial for TIMS performance, and also that all remaining neutral contaminants are moved away from the TIMS entrance. This dual orthogonal design provides robustness against contamination in that neutrals, particles, and droplets are, in two places, driven past the ion optics, into the pumping ports. In studies of this prototype source, we estimate an improvement of a factor of 4.7 in ion transmission, and therefore overall increased signal intensity.

To accommodate the increased ion current, the TIMS analyzer has been updated to a new stacked ring (SRIG) design. This design uses a higher‐order RF field in the ion accumulation region to create a larger effective ion storage volume than the low‐order fields of previous designs. However, a low‐order, quadrupolar field is maintained in the analyzer region to compress the ions toward the analyzer axis during elution to maintain high mobility resolution. In addition, the transition between the high‐order and low‐order portions of the device has been optimized relative to prior designs to further improve performance such as peak shape and resolution under practical conditions. This results in about a factor of 3 gain in ion (charge) capacity and therefore about a factor of 3 in the instrument’s dynamic range.

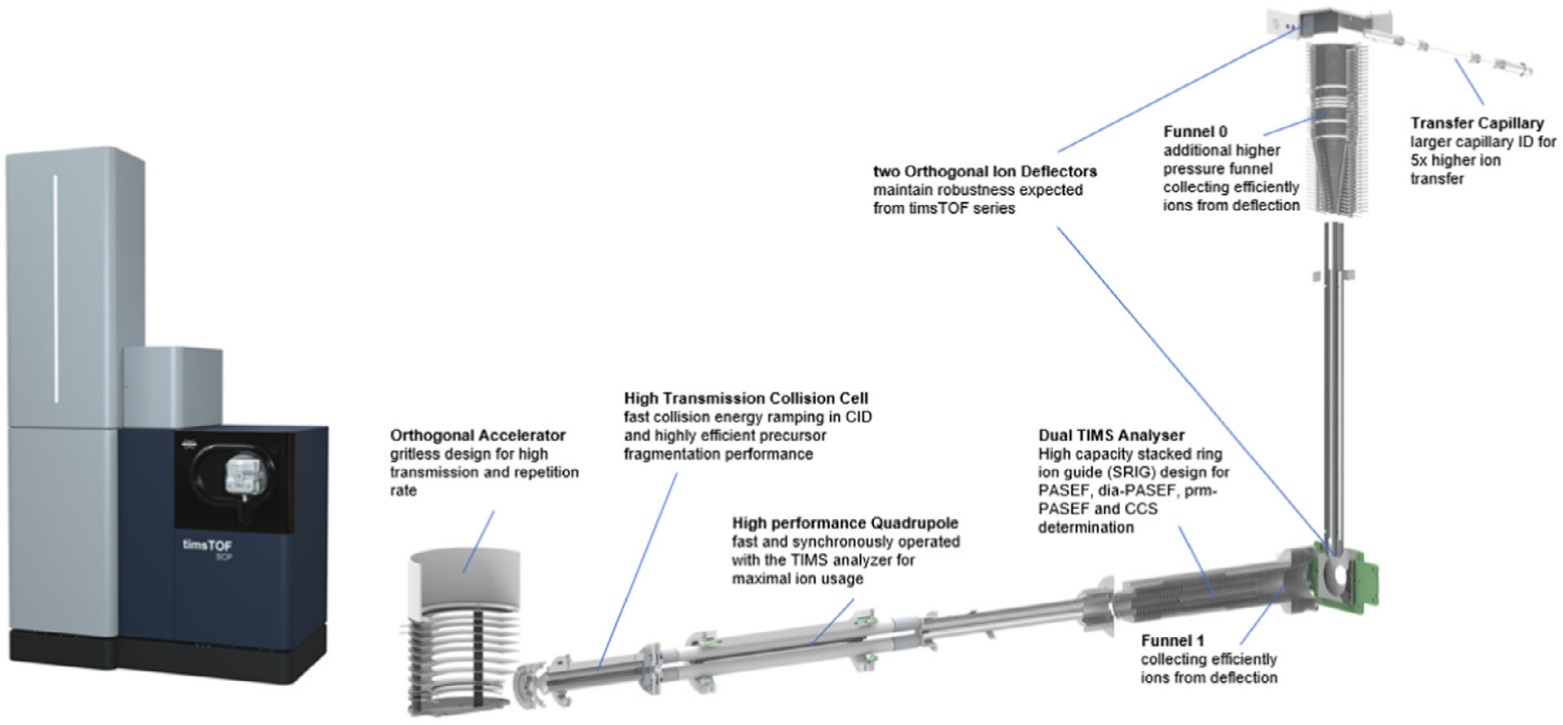

Schematic representation of Bruker timsTOF SCP, which is a high‐performance, ultra‐high sensitivity benchtop mass spectrometer that combines a dual‐TIMS analyzer coupled to a quadrupole, a collision cell that features fast collision energy ramping and a TOF mass analyzer.

#### Mass spectrometry

Mass spectrometric analysis was performed either in a data‐dependent (dda) or data‐independent (dia) PASEF mode. For ddaPASEF, 1 MS1 survey TIMS‐MS and 10 PASEF MS/MS scans were acquired as per acquisition cycle. Ion accumulation and ramp time in the dual TIMS analyzer was set to 50/100/200 ms each and we analyzed the ion mobility range from 1/K_0_ = 1.6 Vs cm^−2^ to 0.6 Vs cm^−2^. Precursor ions for MS/MS analysis were isolated with a 2 Th window for m/z < 700 and 3 Th for m/z > 700 in a total m/z range 100–1,700 by synchronizing quadrupole switching events with the precursor elution profile from the TIMS device. The collision energy was lowered linearly as a function of increasing mobility starting from 59 eV at 1/K_0_ = 1.6 VS cm^−2^ to 20 eV at 1/K_0_ = 0.6 Vs cm^−2^. Singly charged precursor ions were excluded with a polygon filter (otof control, Bruker Daltonik GmbH). Precursors for MS/MS were picked at an intensity threshold of 1,500 arbitrary units (a.u.) and re‐sequenced until reaching a “target value” of 20,000 a.u. considering a dynamic exclusion of 40 s elution. For DIA analysis, we made use of the correlation of ion mobility (IM) with m/z and synchronized the elution of precursors from each IM scan with the quadrupole isolation window. We used the described 100 ms ddaPASEF method for the acquisition of a HeLa bulk single‐shot library for the single‐cell experiments and the short‐gradient diaPASEF method as described in Meier *et al* (2020a), but performed up to five consecutive diaPASEF cycles before the next MS1 scan (see main text). The collision energy was ramped linearly as a function of the IM from 59 eV at 1/K0 = 1.6 Vs cm^−2^ to 20 eV at 1/K0 = 0.6 Vs cm^−2^.

#### Raw data analysis

ddaPASEF data for tryptic HeLa digest dilution series and the cell count experiment were analyzed in the MaxQuant environment (version 1.6.7) and searched against the human Uniprot databases (UP000005640_9606.fa, UP000005640_9606_additional.fa), which extracts features from four‐dimensional isotope patterns and associated MS/MS spectra (Cox & Mann, 2008; Prianichnikov *et al*, 2020). False‐discovery rates were controlled at 1% both on peptide spectral match (PSM) and protein levels. Peptides with a minimum length of seven amino acids were considered for the search, including N‐terminal acetylation and methionine oxidation as variable modifications and cysteine carbamidomethylation as fixed modification, while limiting the maximum peptide mass to 4,600 Da. Enzyme specificity was set to trypsin cleaving C terminal to arginine and lysine. A maximum of two missed cleavages were allowed. Maximum precursor and fragment ion mass tolerance were searched as default for TIMS‐DDA data. Peptide identifications by MS/MS were transferred by matching four‐dimensional isotope patterns between the runs (MBR) with a 0.7‐min retention time match window and a 0.05 1/K_0_ ion mobility window in case of the single‐cell count dilution experiment into a deep ddaPASEF library consisting of 24 fractionations of tryptic HeLa digest. These data were also searched without matching between runs to access the MBR‐mediated identification increase. Either intensity‐based absolute quantification (IBAQ) or label‐free quantification was performed with the MaxLFQ algorithm and a minimum ratio count of 1 (Cox *et al*, 2014).

For all other single‐cell experiments, we used a small library consisting of 25,376 peptides and 4,144 protein groups, which was acquired with the 100 ms ddaPASEF method described above and generated with the MSFRAGGER version 16 using default settings with the exception that cysteine carbamidomethylation was removed from fixed modification (Kong *et al*, 2017; preprint: Demichev *et al*, 2021). All single‐cell measurements were searched against the human UniProt reference proteome (UP000005640_9606.fa and UP000005640_9606_additional.fa) of canonical and isoform sequences.

**Figure EV7:**
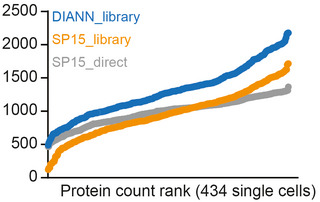
Comparison of single‐cell protein identifications across available DIA software solutions The 434 single‐cell proteome data were processed with either DIA‐NN or Spectronaut using a spectral library, or using Spectronaut in directDIA mode. Protein identifications were plotted as a function of the protein rank for each single cell.

Due to recent software improvement driven by the implementation of the next generation of Spectronaut 15, followed by DIA‐NN 1.8 (Bruderer *et al*, 2015; preprint: Demichev *et al*, 2021) for the analysis of diaPASEF raw files, which utilize the complex diaPASEF TIMS‐TOF data much better by improved machine learning algorithms, we initially evaluated both software solutions for the analysis of our single‐cell data set. It turned out that DIA‐NN 1.8 using spectral libraries generated with MSFRAGGER, at that time, outperformed the library‐based and directDIA analysis pipeline in Spectronaut 15 in our hands. This is consistent with the reports of DIA‐NN being used for sample‐limited analyses and very short gradients (Messner *et al*, 2020, 2021; preprint: Demichev *et al*, 2021; Fig EV7).

According to the DIA‐NN authors, this benefit results from the advanced use of deep learning algorithms for signal deconvolution and for diaPASEF data specifically from the additional ion mobility resolution that makes full use of the correlation between ion mobility and m/z on MS2 level for fragment signal pattern identification and its matching to the precursor level. That said, our main focus in the current manuscript was not to present computational advances and a benchmarking of DIA‐NN versus Spectronaut, but to highlight our scalable and robust MS‐based single‐cell proteomics workflow. Furthermore, because of its somewhat better performance and to present a unified analysis, we consistently analyzed all data with DIA‐NN.

Raw files were analyzed with DIA‐NN (version 1.8; Demichev *et al*, 2020) using default settings (e.g., 1% precursor and protein FDR), except changing the covered peptide length range to 7–50, precursor charge range to 2–4, enabling MBR, turning of protein inference to use the inference from the library generated by MSFRAGGER, quantification strategy to robust LC (high precision), and library generation to IDs, RT, and IM profiling.

#### Visualization and FDR estimates of fragment ion intensities

Quantitative fragment ion profiles were generated from the DIA‐NN output table. Only fragment ions used for quantification in DIA‐NN were included. To cancel out cell size‐dependent abundance changes, one normalization factor was estimated per cell, using fold‐change‐based normalization of the whole data set, as described in the MS‐EmpiRe method, which we also used for FDR control (Ammar *et al*, 2019). The intensities were log_2_ transformed and subsequently visualized.

#### Proteomics downstream data analysis

**Figure EV8:**
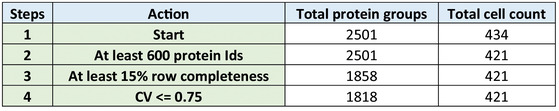
Single‐cell data filtering for processing The 434 single‐cell proteome data were filtered first for at least 600 protein identifications, then for at least 15% data completeness across rows and finally for coefficients of variation of below 0.75 before the downstream processing and biological interpretation was performed.

Proteomics data analysis was performed in the Perseus environment (version 1.6.7, 1.5.5) (Tyanova *et al*, 2016), GraphpadPrism (version 8.2.1), and Python (version 3.8.2). MaxQuant output tables were filtered for “Reverse,” “Only identified by site modification,” and “Potential contaminants” before further processing. Ontologies for the biological process and cellular compartment assignment for proteins were performed with the mainAnnot.homo_sapiens.txt.gz, followed by categorical counting across all proteins for each of the ontologies, and counts were exemplary visualized as frequency plot. For single‐cell analysis, if not otherwise specified, the DIA‐NN protein group data output was filtered first for at least 600 protein observations per cell and at least 15% quantification events across rows and log(x + 1) transformed resulting in the following cell numbers and protein quantifications (Fig EV8).

For correlation analysis of two protein expression vectors, transformed gene or protein quantification events of two cells were plotted against each other replacing missing values by zeros. For principal component analysis (PCA), missing values were imputed from a normal distribution with a width of 0.3 standard deviations that was downshifted by 1.8 standard deviations. Differential expression analysis by two‐sided unpaired *t*‐test was performed on two groups filtered for at least 50% row‐wise quantification events within one group. False discovery rate control due to multiple hypothesis testing was performed by a permutation‐based model and SAM statistic with an S_0_ parameter of 0.3. For cell size estimation based on raw MS signal, intensity outputs within cell cycle resolved single‐cell proteomics results were summed up and visualized as boxplots. The core proteome was calculated by filtering the whole single‐cell proteomics data set for at least 70% quantification events for each protein followed by selection of the top 200 proteins with the smallest coefficient of variation across the data set.

#### Single‐cell protein and RNA comparison and dropout statistics

The SMART‐Seq2 (Hu *et al*, 2019) data set measured 720 HeLa cells in three different batches with a total of 24,990 expressed genes. The Drop‐seq (Schwabe *et al*, 2020) data set contained three batches with a total of 5,665 cells and 41,161 expressed genes. We performed the single‐cell analysis with scanpy v1.6.0 (Wolf *et al*, 2018). If not stated otherwise, we used standardized filtering across all data sets, removed cells with less than 600 genes expressed, and removed genes detected in < 15% of the remaining cells, resulting in 10,557 transcripts in 720 cells in the SMART‐Seq2 data set and 5,022 transcripts and 6,701 cells measured with Drop‐seq technology. Ratios of non‐zero entries in the scRNAseq data sets and the number of identified proteins in our data are summarized as violin plots. To investigate data completeness across covered dynamic range, we computed the data completeness as a function of the mean log(x + 1)‐transformed protein abundance of all non‐zero/‐NaN entries. We included the expected data completeness based on the assumption that missing values are purely due to shot‐(Poisson)‐noise as 1‐exp(‐x). For correlation analysis, the RNA abundance entries were linearly scaled to sum to the mean cell size of the respective data set per cell (231,281.56 for SMART‐Seq2 and 7,808.12 for Drop‐Seq) followed by log(x + 1) transformation of all abundance entries. Correlation values between the expressions of two cells were computed as the Pearson correlation on the 1,672 genes that were shared in all three data sets. Entries of missing protein abundance values were excluded from the specific computation. For the PCA plot of technological comparisons, the gene coverage intersection of all technologies (1,672) was isolated, NaNs were replaced by zeros, and expression values were linearly scaled to 1E6 followed by log(1 + x) transformation. In coefficient of variation (CV) versus CV plots comparing different technologies as well as the mean versus CV analysis (including the core proteome analysis) and the CV distribution boxplots, RNA expression vectors were scaled to the mean cell size of that measurement technology and mean and CV values were computed per gene under the assumption that single‐cell RNA‐sequencing data are not zero inflated (Svensson, 2020) while NaNs were excluded for the proteomics data. CV (Proteomics) versus CV (RNA‐seq) plots show the comparison of CV values of proteins/genes that were shared between all data sets.

#### Cell cycle state prediction

Cell cycle predictions were performed using the scanpy method score_genes (Wolf *et al*, 2018) based on three sets of proteins that are specifically expressed in the G1 (MARCKS, KRT1, HIST1H1E, KRT18, HNRNPA1, CHCHD3, CD44, NASP, TARDBP, PODXL, SUMO2, STMN1, TRIM28, and SPTAN1), S (NOLC1, ATP2A2, CANX, TMX1, CKB, SLC25A3, SLC16A1, MT‐CO2, SRPRB, CYB5R3, LETM1, and ANP32B), or G2/M phase (TOP2A, HMGB1, EIF5B, TMSB10, EIF3D, ANP32A, RCC2, FASN, LUC7L2, AARS, KPNA2, and CKAP5), respectively. The cell phase‐specific protein sets were selected based on the z‐scored fold‐change ratios provided in Geiger and coworkers (Aviner *et al*, 2015). The top 60 highest differentially expressed genes were selected and filtered for quantification in at least 70% of our cells. This scoring method yields the average expression on the provided set of genes minus the average expression on a reference set of genes for each cell. The reference set is chosen to mirror the average expression of the target gene set. For this analysis, cells were filtered, log(x + 1) transformed, and missing values replaced by zeros. Plotted are the ROC curves for the three scores corresponding to the three sets of characteristic proteins (G1, S, and G2 M) used individually to discriminate between the cells of two cell cycle stages.

## Author contributions

**Andreas‐David Brunner:** Conceptualization; Data curation; Formal analysis; Validation; Investigation; Visualization; Methodology; Writing—original draft; Project administration; Writing—review and editing. **Marvin Thielert:** Data curation; Formal analysis; Investigation; Methodology. **Catherine Vasilopoulou:** Data curation; Formal analysis; Investigation; Methodology. **Constantin Ammar:** Data curation; Software; Formal analysis; Investigation; Methodology. **Fabian Coscia:** Resources; Investigation; Methodology. **Andreas Mund:** Resources; Investigation; Methodology. **Ole B Hoerning:** Resources; Software; Methodology. **Nicolai Bache:** Resources; Software; Investigation; Methodology. **Amalia Apalategui:** Resources; Software; Methodology. **Markus Lubeck:** Resources; Software; Investigation; Methodology. **Sabrina Richter:** Data curation; Software; Formal analysis; Investigation; Visualization; Methodology. **David S Fischer:** Conceptualization; Software; Formal analysis; Validation; Investigation; Visualization; Methodology. **Oliver Raether:** Conceptualization; Resources; Software; Formal analysis; Supervision; Investigation; Methodology; Project administration. **Melvin A Park:** Resources; Methodology. **Florian Meier:** Conceptualization; Resources; Supervision; Investigation; Methodology. **Fabian J Theis:** Conceptualization; Resources; Software; Formal analysis; Supervision; Funding acquisition; Investigation; Methodology. **Matthias Mann:** Conceptualization; Resources; Supervision; Funding acquisition; Investigation; Methodology; Writing—original draft; Project administration; Writing—review and editing.

In addition to the CRediT author contributions listed above, the contributions in detail are:

AD‐B and MM conceptualized and designed the study. AD‐B, MT, FC, and AM performed experiments. MAP and OR designed the new mass spectrometer. AD‐B, OBH, and NB conceived the new EvoSep gradient. OBH, NB, AD‐B, and MT designed the new EvoSep gradient and optimized it for proteomics performance. DSF and FJT conceptualized the single‐cell modeling. AD‐B, SR, DSF, FJT, CA, MT, OBH, NB, CV, AA, ML, FM, and MM analyzed the data. AD‐B and MM wrote the manuscript.

## Disclosure statement and competing interests

ML, OR, AA, and MAP are employees of Bruker Daltonik. OBH and NB are employees of EvoSep Biosystems. MM is an indirect shareholder in EvoSep Biosystems. FJT reports receiving consulting fees from Roche Diagnostics GmbH and Cellarity Inc., and ownership interest in Cellarity Inc. and Dermagnostix. All other authors have no competing interests.

## Supporting information



Expanded View Figures PDF

Dataset EV1

Dataset EV2

Dataset EV3

Dataset EV4

## Data Availability

All mass spectrometry raw data, libraries, and outputs from each particular search engine analyzed in this study have been deposited to the ProteomeXchange Consortium via the PRIDEpartner repository. Project accession: PXD024043 (www.ebi.ac.uk/pride/archive?keyword=PXD024043). The code used for data analysis can be found as a Jupyter Notebook at: https://github.com/theislab/singlecell_proteomics.
