# Supplementary material for: Ultra‐high sensitivity mass spectrometry quantifies single‐cell proteome changes upon perturbation
Source: Mol Syst Biol. 2022 Feb 28;18(3):e10798. doi: 10.15252/msb.202110798 (PMC8884154; doi:10.15252/msb.202110798)
Supplement: Supplementary file 1 — Expanded View Figures PDF [file MSB-18-e10798-s003.pdf]

Expanded View Figures

Figure EV1. Technical feasibility assessment of ultra-high sensitivity mass spectrometry and liquid chromatography.

A Ranked protein identifications for six-cell measurements with and without matching between runs. Zero-cell protein identifications are highlighted in orange and overlaid on the six-cell protein rank plot. The top 10 protein identifications of the zero-cell runs are depicted.

B True nanoflow at 25, 50, and 100 nl/min flow rate on the EvoSep One liquid chromatography system.

C Standardized 100 nl/min true nanoflow gradient on the EvoSep One liquid chromatography system. Pressure (Left) and flow profile (right) of the gradient of more than 1,000 consecutive runs (Day 1–Run #1 = gray; Day 20–Run #500 = orange; and Day 45–Run #1,000 = blue).

D Data completeness (Blue) and coefficient of variation (Orange) evaluation of different diaPASEF consecutive scan repetitions merged for the analysis of 1 ng tryptic HeLa digest. Scans were varied from one, three, and five repetitions.

 © 2022 The Authors

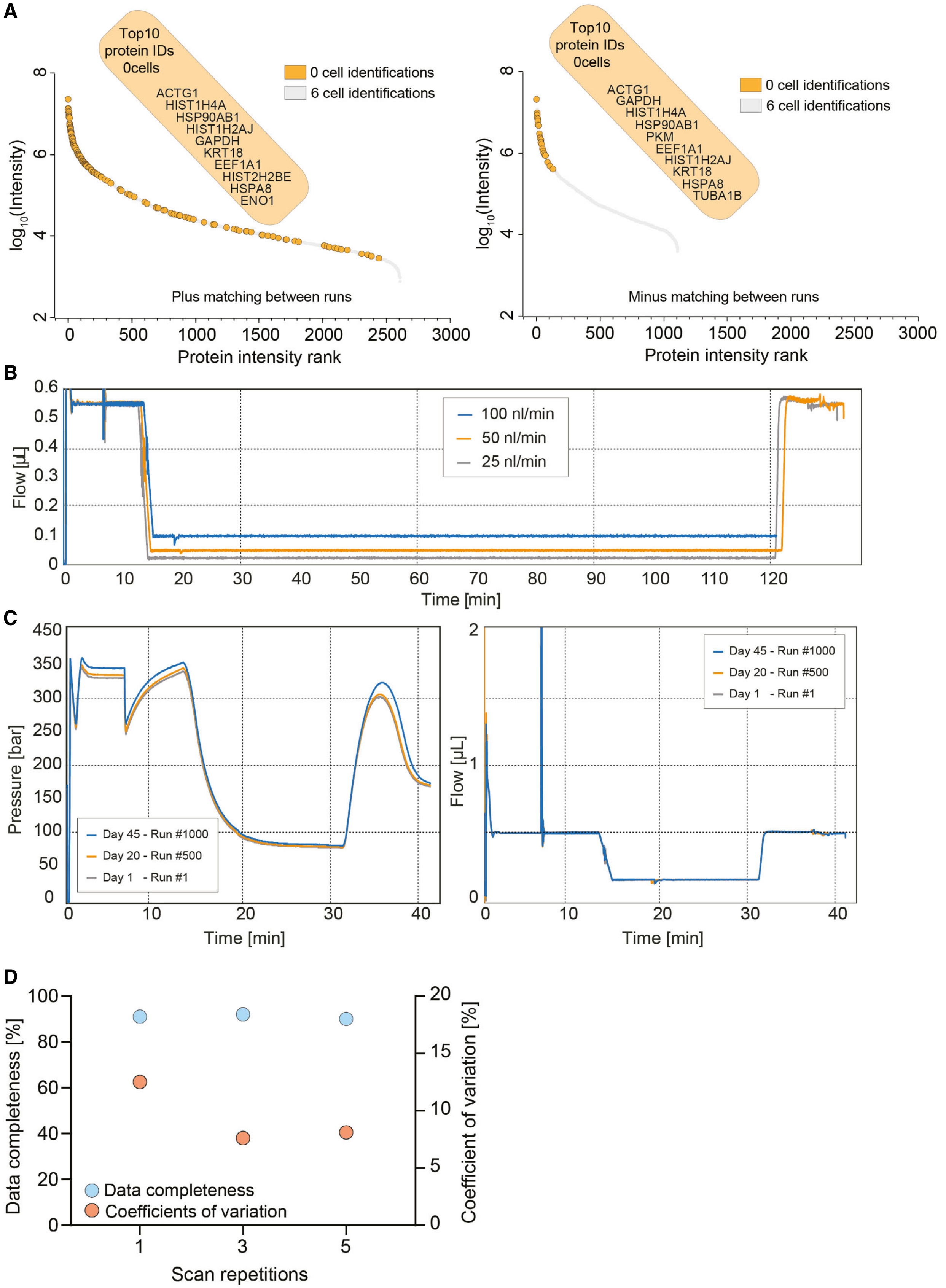
A
Top10 protein IDs 0cells
ACTG1
HIST1H4A
HSP90AB1
HIST1H2AJ
GAPDH
KRT18
EEF1A1
HIST2H2BE
HSPA8
ENO1
0 cell identifications
6 cell identifications
log10(Intensity)
Plus matching between runs
Protein intensity rank
Top10 protein IDs 0cells
ACTG1
GAPDH
HIST1H4A
HSP90AB1
PKM
EEF1A1
HIST1H2AJ
KRT18
HSPA8
TUBA1B
0 cell identifications
6 cell identifications
log10(Intensity)
Minus matching between runs
Protein intensity rank
B
100 nl/min
50 nl/min
25 nl/min
Flow [µL]
Time [min]
C
Pressure [bar]
Day 45 - Run #1000
Day 20 - Run #500
Day 1 - Run #1
Time [min]
Day 45 - Run #1000
Day 20 - Run #500
Day 1 - Run #1
Flow [µL]
Time [min]
D
Data completeness [%]
Coefficient of variation [%]
Data completeness
Coefficients of variation
Scan repetitions

Figure EV1.

Figure EV2. True single-cell proteomics data set description.

A Frequency plot for coefficient of variation occurrence within the 420 single-cell proteomics data set.
B Protein $\log_{10}$ intensity versus coefficient of variation.
C Raw log(x + 1)-transformed intensity values of proteins per cell plotted against the number of identified proteins per cell (Left) and after normalization by local regression to cancel out those differences to enable downstream analysis (Right).
D Principal component analysis of cell cycle stage enriched single-cell proteomics measurements and three cell culture batches projected on top.
E Category count of gene ontology annotations for cellular compartment and biological process terms. Exemplary, category count terms are shown for the cellular compartment (Left) and biological process (Right) for more than 430 single-cell proteomics data set.
F Cell cycle stage prediction for G2 versus G1 phase cells (Left) and G2/M versus G1 phase cells (Right) using the 60 topmost differentially expressed proteins reported by Geiger and coworkers (Aviner *et al*, 2015) as input.

 © 2022 The Authors

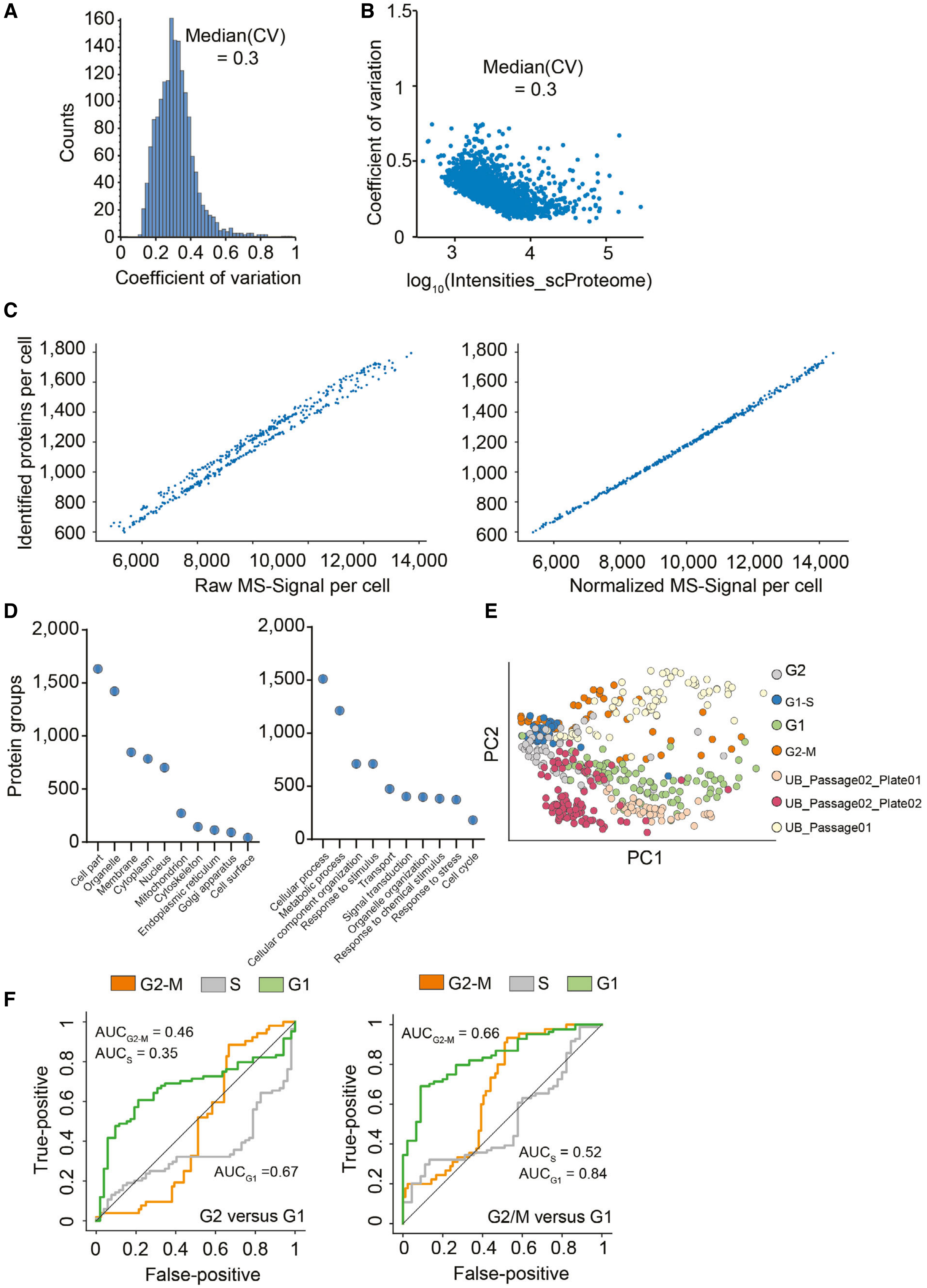
A
Median(CV) = 0.3
Counts
Coefficient of variation
B
Median(CV) = 0.3
Coefficient of variation
log10(Intensities_scProteome)
C
Identified proteins per cell
Raw MS-Signal per cell
Normalized MS-Signal per cell
D
Protein groups
Cell part
Organelle
Membrane
Cytoplasm
Nucleus
Mitochondrion
Cytoskeleton
Endoplasmic reticulum
Golgi apparatus
Cell surface
Cellular process
Metabolic process
Cellular component organization
Response to stimulus
Transport
Signal transduction
Organelle organization
Response to chemical stimulus
Response to stress
Cell cycle
E
PC2
PC1
G2
G1-S
G1
G2-M
UB_Passage02_Plate01
UB_Passage02_Plate02
UB_Passage01
F
G2-M
S
G1
AUCG2-M = 0.46
AUCS = 0.35
AUCG1 =0.67
G2 versus G1
True-positive
False-positive
AUCG2-M = 0.66
AUCS = 0.52
AUCG1 = 0.84
G2/M versus G1

Figure EV2.

Figure EV3. Peptide fragment ion intensities of several proteins.

Fragment ion intensities of peptides for several differentially expressed proteins (HDAC2, FDR = 1.2E-3; UBE2C, FDR = 4.7E-6; UBE2S, FDR = 4.1E-15; TMSB10, FDR = 4.1E-15; BCCIP, FDR = 4.7E-1; HMGA1, FDR = 1.4E-2; NACA, and FDR = 4.1E-15) in the comparison of nocodazole- (G2-M transition) and thymidine (G1-S transition)-treated cells. Boxplots represent the intensity distribution of indicated peptide fragment ion intensities.

 © 2022 The Authors

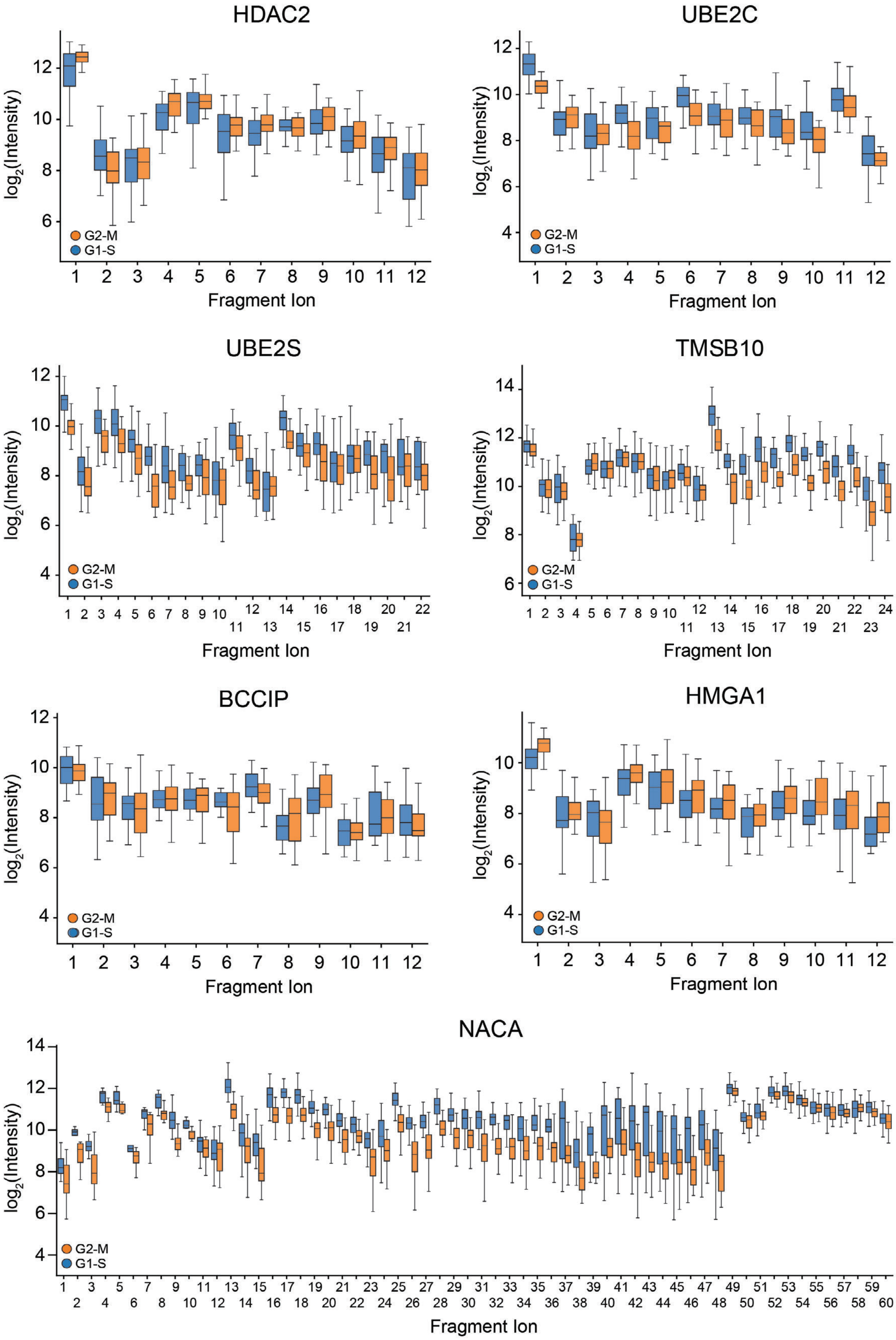
HDAC2
UBE2C
UBE2S
TMSB10
BCCIP
HMGA1
NACA
log2(Intensity)
Fragment Ion
G2-M
G1-S

Figure EV3.

Figure EV4. Correlation and gene/protein completeness analysis of single-cell transcriptome sequencing and our LC–MS-based single-cell proteomics data set of the same cell line.

A Pearson correlation of observations for each cell within each of the technologies on all genes (MS-based proteomics, SMART-Seq2 RNA sequencing, and droplet-based RNA sequencing; Left) and for each cell within each of the technologies on shared genes between technologies (MS-based proteomics, SMART-Seq2 RNA sequencing, and droplet-based RNA sequencing; Right).

B Gene/Protein expression completeness per cell on all shared genes between the three technologies (scProteomics; SMART-seq2; and DROP-seq).

C Gene and protein expression completeness as a function of ranked genes/proteins for all three technologies (Proteomics, DROP-seq, and SMART-Seq2). Arrows indicate a bimodal distribution for single-cell RNAseq data in both technologies, which is absent in proteomics.

D Data completeness across single cells as a function of mean protein abundance for MS-based single-cell proteomics and both single-cell RNA sequencing (Drop-Seq, SMART-Seq2). Expected poison dropout distribution shown in red.

E Scatter plot of two independently measured single-cell proteome expression values.

© 2022 The Authors

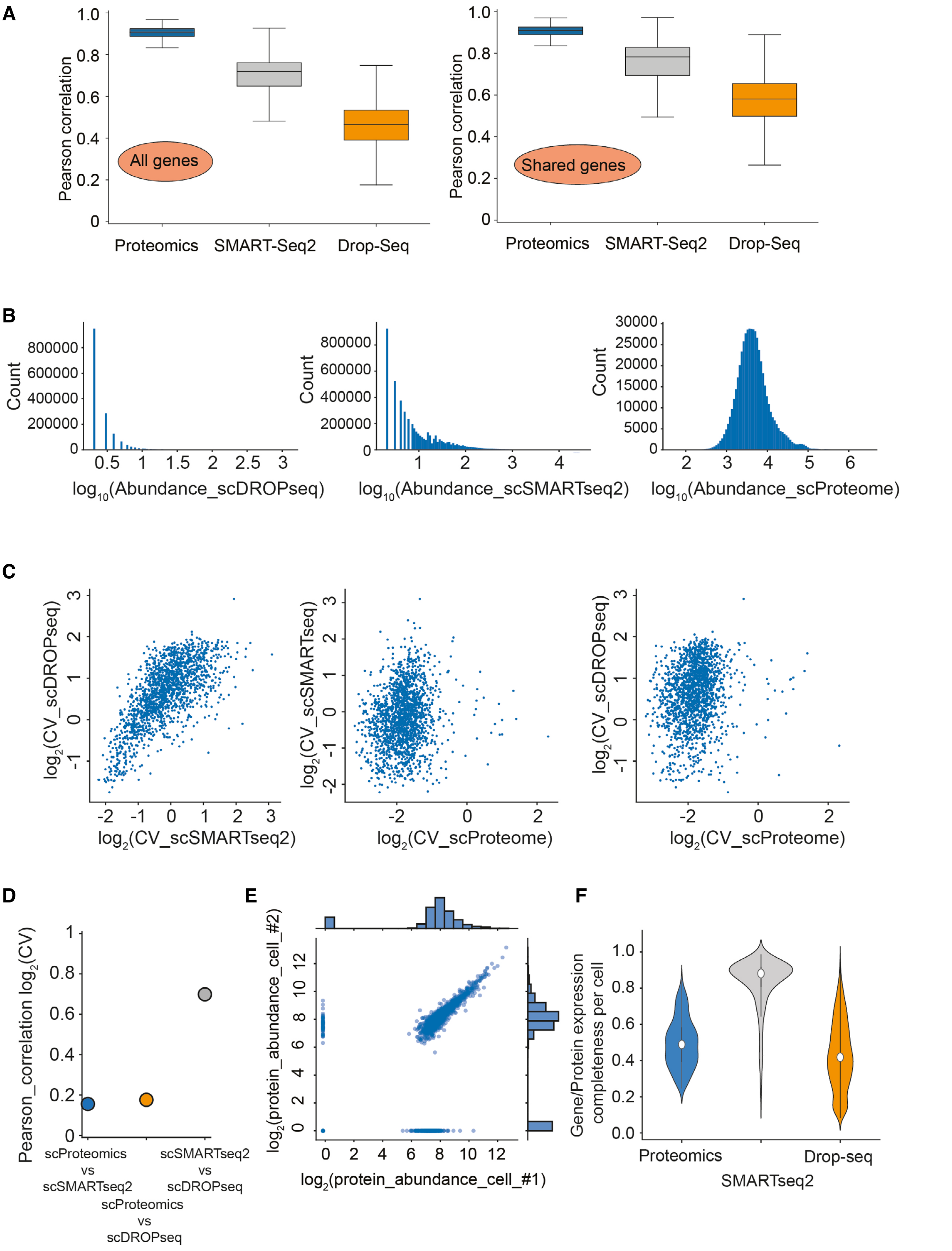
A
Pearson correlation
All genes
Shared genes
Proteomics
SMART-Seq2
Drop-Seq
B
Count
log10(Abundance_scDROPseq)
log10(Abundance_scSMARTseq2)
log10(Abundance_scProteome)
C
log2(CV_scDROPseq)
log2(CV_scSMARTseq2)
log2(CV_scSMARTseq)
log2(CV_scProteome)
D
Pearson_correlation log2(CV)
scProteomics vs scSMARTseq2
scProteomics vs scDROPseq
scSMARTseq2 vs scDROPseq
E
log2(protein_abundance_cell_#2)
log2(protein_abundance_cell_#1)
F
Gene/Protein expression completeness per cell
Proteomics
SMARTseq2
Drop-seq

Figure EV4.

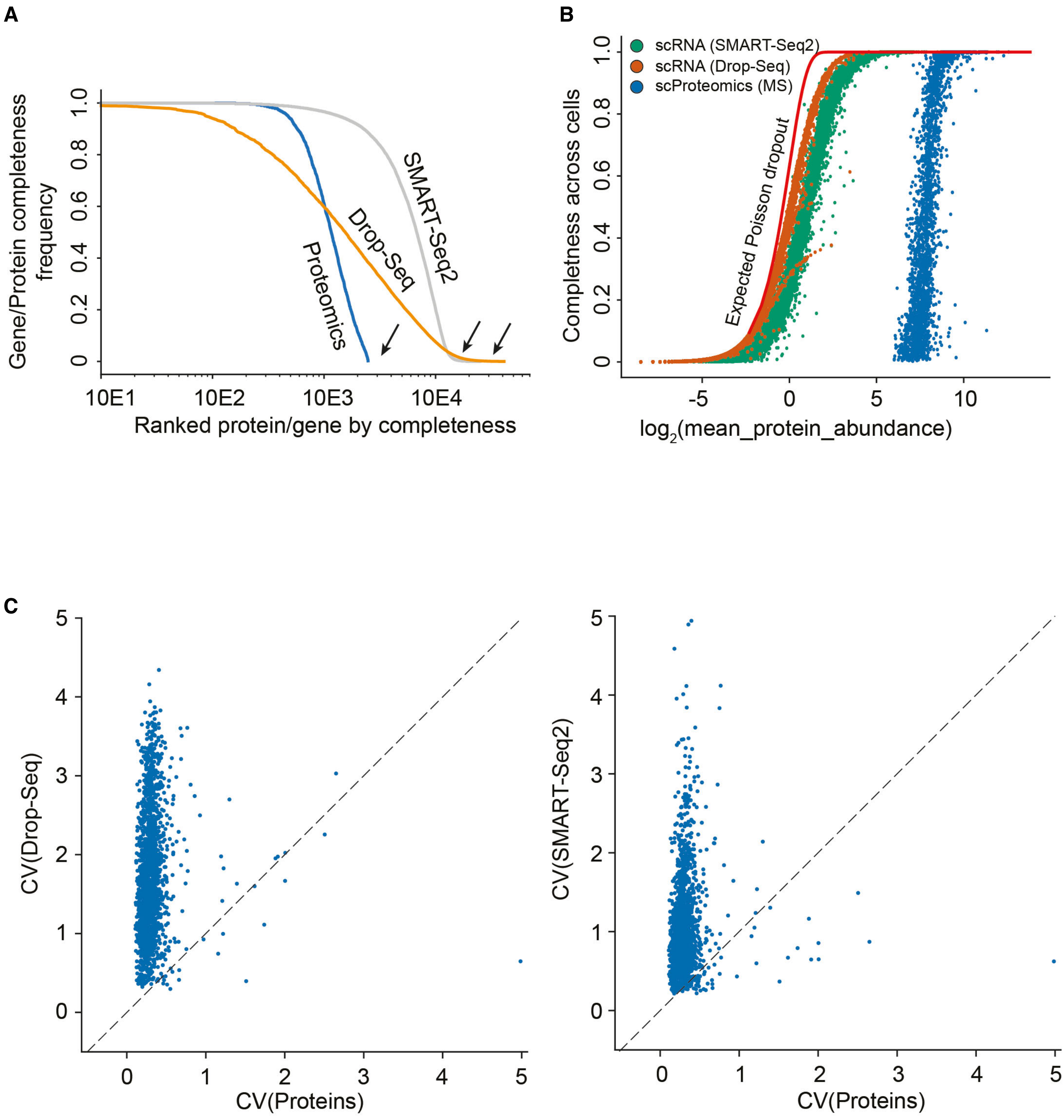

Figure EV5. Data distribution analysis of single-cell transcriptome sequencing and our LC–MS-based single-cell proteomics data set of the same cell line.

A Histogram of $\log_{10}$ abundance of scDROPseq (left), scSMARTseq2 (middle), and scProteomics data (right).

B The coefficient of variation of a gene measured by either Drop-Seq technology (Left) or SMART-Seq2 (Right) compared to the coefficient of variation of the corresponding protein measured by MS-based single-cell proteomics.

C Pearson correlation of coefficients of variation for each gene shared within each comparison.

 © 2022 The Authors

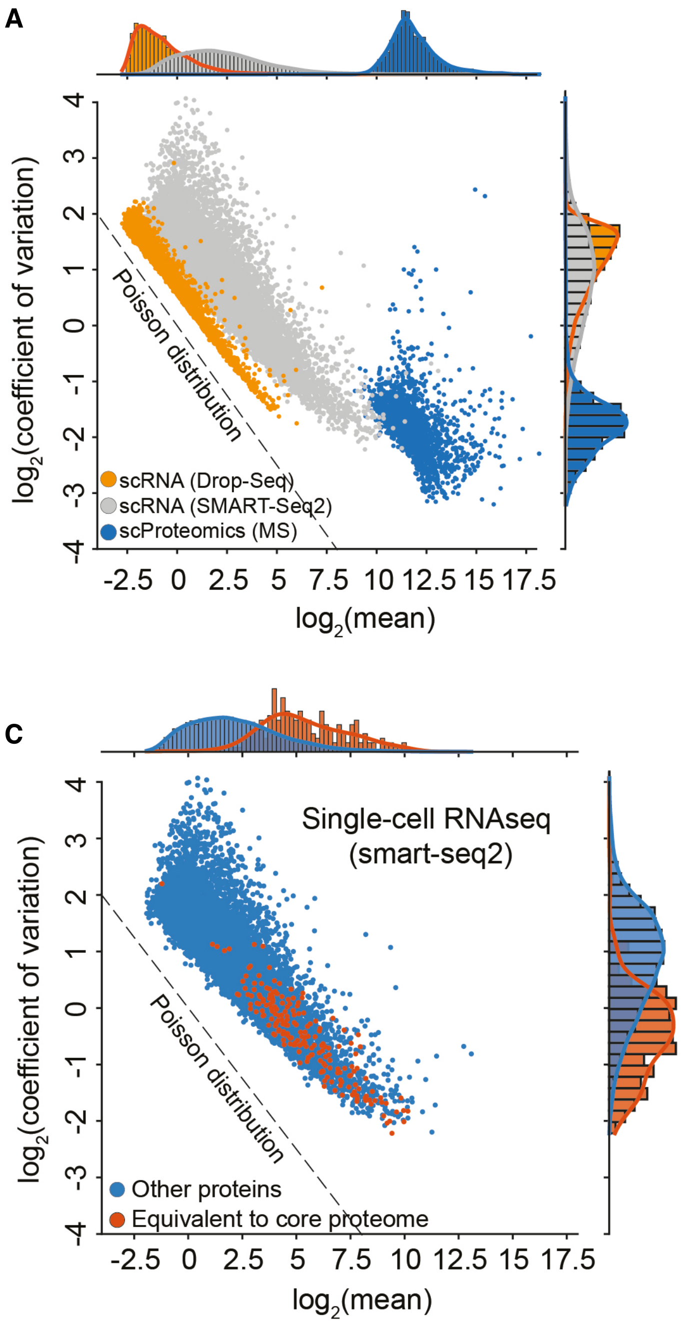

Figure EV6. Single-cell core proteome distribution compared to matched gene expression distribution in single-cell transcriptome data of the same cell line.

A Coefficient of variation distribution as a function of $\log_2$ mean gene or protein intensities for Drop-Seq (Orange), SMART-Seq2 (Gray), or MS-based single-cell proteomics (Blue). Expected Poisson distribution shown as dashed line.

B Coefficient of variation of single-cell RNA-sequencing (drop-seq) levels as a function of mean expression levels with the "core proteome" colored in orange and non-"core proteome" genes in blue. Expected Poisson distribution shown as dashed line.

C Coefficient of variation of single-cell RNA-sequencing (smart-seq2) levels as a function of mean expression levels with the "core proteome" colored in orange and non-"core proteome" genes in blue. Expected Poisson distribution shown as dashed line.

© 2022 The Authors

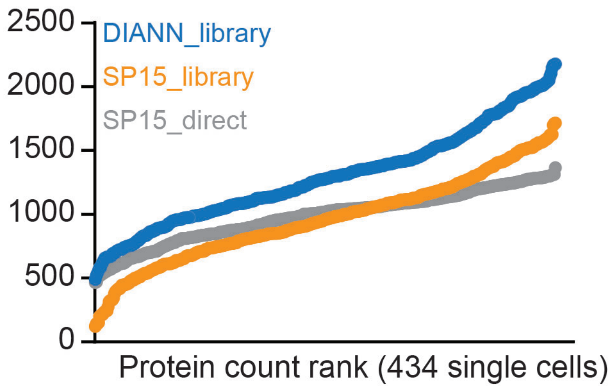

Figure EV7. Comparison of single-cell protein identifications across available DIA software solutions.

The 434 single-cell proteome data were processed with either DIA-NN or Spectronaut using a spectral library, or using Spectronaut in directDIA mode. Protein identifications were plotted as a function of the protein rank for each single cell.

Steps	Action	Total protein groups	Total cell count
1	Start	2501	434
2	At least 600 protein Ids	2501	421
3	At least 15% row completeness	1858	421
4	CV <= 0.75	1818	421

Figure EV8. Single-cell data filtering for processing.

The 434 single-cell proteome data were filtered first for at least 600 protein identifications, then for at least 15% data completeness across rows and finally for coefficients of variation of below 0.75 before the downstream processing and biological interpretation was performed.

 © 2022 The Authors
