# Supplementary material for: Ultra‐high sensitivity mass spectrometry quantifies single‐cell proteome changes upon perturbation
Source: Mol Syst Biol. 2022 Feb 28;18(3):e10798. doi: 10.15252/msb.202110798 (PMC8884154; doi:10.15252/msb.202110798)
Supplement: Supplementary file 6 [file MSB-18-e10798-s005.pdf]

molecular systems biology

Ultra-high sensitivity mass spectrometry quantifies single-cell proteome changes upon perturbation

Andreas-David Brunner, Marvin Thielert, Catherine Vasilopoulou, Constantin Ammar, Fabian Coscia, Andreas Mund, Ole Hoerning, Nicolai Bache, Amalia Apalategui, Markus Lubeck, Sabrina Richter, David Fischer, Oliver Raether, Melvin Park, Florian Meier, Fabian Theis, and Matthias Mann
DOI: 10.15252/msb.202110798

Corresponding author(s): Matthias Mann (mmann@biochem.mpg.de)

Review Timeline:

Submission Date:	8th Nov 21
Editorial Decision:	30th Nov 21
Revision Received:	22nd Dec 21
Editorial Decision:	1st Feb 22
Revision Received:	7th Feb 22
Accepted:	8th Feb 22

Editor: Maria Polychronidou

Transaction Report:

(Note: With the exception of the correction of typographical or spelling errors that could be a source of ambiguity, letters and reports are not edited. Depending on transfer agreements, referee reports obtained elsewhere may or may not be included in this compilation. Referee reports are anonymous unless the Referee chooses to sign their reports.)

Thank you again for submitting your work to Molecular Systems Biology. We have now heard back from two of the three reviewers who agreed to evaluate your study. In the interest of time, I decided to send you an early decision based on these two reports so that you can start performing the revisions. When we receive the comments from reviewer #3 (they promised to send them early this week), I will forward them to you. As you will see below, the reviewers think that the study is a valuable contribution to the single-cell proteomics field. They raise however a series of concerns, which we would ask you to address in a revision.

I think that the reviewers' recommendations are clear and therefore there is no need to repeat the points listed below. The reviewers mainly raise technical concerns and recommend including further discussion to better contextualize the study with respect to previous work in the field. All issues raised by the reviewers need to be satisfactorily addressed. Please contact me in case you would like to discuss in further detail any of the issues raised.

On a more editorial level, we would ask you to address the following points:

Reviewer #1:

Summary

In their manuscript, Brunner et al. present a single-cell proteomics workflow that is facilitated by multiple significant advances in the field. The novel mass spectrometer presented (Bruker timsTOF SCP) shows a clear improvement in terms of sensitivity, which, in combination with the diaPASEF acquisition method and a novel implementation of the EvoSep One LC system, leads to great results in terms of proteome coverage of single cells. Especially when analyzed by sophisticated machine-learning based spectral interpretation engines (e.g. DIA-NN), the authors appear to reach unprecedented proteome depth in label-free single cells. Also in terms of cell throughput, the authors set a new standard (40 cells per day), although when put in the context of multiplexed approaches, their throughput arguably still falls short. Nevertheless, through a series of experiments focusing on manipulating the cell cycle in HeLa cells, the authors demonstrated a "core proteome" to exist, especially compared to transcriptome-level readouts which appear to be more stochastic. Overall, the authors demonstrate the power of teaming up with top-level industry partners, and provide an additional tool in the arsenal of newly established single cell proteomics by Mass Spectrometry (SCP) approaches.

General remarks

Overall, the key conclusions made throughout the manuscript seem generally sound, however, the authors on several occasions appear selective in placing their work in context of previous SCP work, which is somewhat problematic. For example in the opening paragraph, they end with claiming that "a technology that provides quantitatively accurate MS proteomics data from true single cell-derived signals (T-SCP) and answers biological questions is still outstanding". In my opinion, this does not do justice to efforts made on e.g. the NanoPots platform by Ryan Kelly, Ying Zhu and colleagues or alternative chip-systems by Catherine Wong et al, who have collectively absolutely pioneered taking MS based approaches to the single cell level, and have demonstrated their utility in a variety of biological questions. The main benefit of the approach presented here is the significantly improved cell throughput while keeping proteome depth high, and this is a significant development in its own right that deserves to be the main selling point in my opinion.

The authors opt to apply a technology (single cell approaches) originally developed for detecting cellular heterogeneity to a homogeneous cell line, into which they introduce heterogeneity through a variety of cell cycle inhibitors. The exact biological impact of this particular investigation, beyond comparing it to transcriptome level readouts, still somewhat eludes me, and I am not convinced that in terms of cell cycle regulation at the protein level, that any new biological insights were generated compared to previous bulk-cell level investigations. Perhaps more cells would be needed to truly detect distinct cell states within their enriched populations, but this is not really discussed in the manuscript. If the authors' main intention was to demonstrate the ability of their SCP workflow to read-out different cell states, then this was successful.

The observations about the core proteome, especially when compared to the transcriptome are very interesting and provide some novel insights into molecular cell biology with single cell resolution. It was interesting that they spanned the covered dynamic range of the proteome, and that the corresponding CVs at the transcriptome level did not suggest such a high level of stability. This work as a whole is a clear example of how MS-based proteomics provides a complementary view to RNAseq, and now also at the single cell level. As pointed out by the authors, these core proteins could represent appropriate loading controls on e.g. western blot or PRM analysis and was a neat spin-off conclusion to their data exploration efforts.

Major points

As described above, the authors present a convincing experimental workflow which lifts label-free quantification of protein levels using MS to the next level. However, I find that the manuscript falls short in the evaluation of the quantitative accuracy of the up to 2000 proteins measured in some of the single-cells. Furthermore, I have multiple concerns about the comparison of the measured proteome to the measured transcriptome as detailed below:

On page 4 and figure 2C & D, the authors claim high quantitative accuracy by comparing abundance values of proteins between measurements. However, this analysis only evaluates the quantitative precision across the intensity range and reveals that precision is highest at high abundance and decreases towards lower abundances. The authors should evaluate the quantitative accuracy of their readout by comparing measured fold-changes on single-cell level to fold-changes measured to bulk data (i.e. approximation of the ground truth); this analysis has been performed extensively for SCoPE-MS experiments around the world. Alternatively, quantitative accuracy could be evaluated by providing ground-truth fold-changes in the experimental design e.g. by spiking in known peptide amounts/ratios. I hypothesize that quantitative accuracy is highly dependent on signal intensity, and thus, it would important to investigate how many of the up to 2000 proteins provide a reliable quantitative readout and can be used for biological interrogation. The authors repeat that mis-interpretation on page 8 and Extended data Fig. 2D, by presenting the coefficient of variation (CV), which is a measure of precision, and not quantitative accuracy, as claimed in the text.

On page 10 and Extended data Fig. 4A, the authors claim that cells have higher correlation on average than in the droplet-based method and similar correlation to the SMART-Seq2 method. However, I assume that this analysis was done with all genes measured [T-SCP (Cells x Proteins: 424 x 2,480), SMARTseq2 (Cells x Genes: 720 x 24,990), and Drop-seq (Cells x Genes: 5,383 x 41,161)]. Thus, the trend of the strength of correlation follows the amount of genes measured, and therefore could simply reflect the decreased number of reads per gene, and thus higher measurement noise, if more genes are measured. The authors should compare the correlation of the cells using the same genes/proteins across the datasets or take the top 2,480 genes in the rna-seq datasets to compare to the 2,480 proteins.

Similarly, on page 10 and Fig. 5A, the authors show decreased data completeness for the sc-RNAseq methods. However, these methods also measured many more genes. For a fair comparison, the authors should compare the data completeness of the same genes/proteins across the datasets or take the top 2,480 genes in the rna-seq dataset to compare to the 2,480 proteins. Comparing only these subsets could also change the observation of bimodal gene completeness frequency distribution in sc-RNAseq in Extended data Fig. 4b.

On page 10/11 and Fig. 5B, the authors show that protein measurements separate strongly from RNA in a principal component analysis. I'm a bit concerned that this observation could be biased by including missing values in the comparison, as the underlying reason for missing values could be technology dependent. I suggest to repeat the analysis with a smaller subset of cells and overlapping genes that are completely quantified in all methods. Alternatively, missing values could be imputed, instead of replacing them with 0. Furthermore, I would suggest to scale the data to zero mean and standard deviation 1 to remove potential bias of different dynamic ranges of the different data types. Finally, if the results turn out to be similar, I would suggest to investigate which genes drive the separation of the protein data from the RNA data in principal component 2, as this would be of great biological interest.

On page 11 and Fig. 5c, the authors claim that single-cell transcript expression levels correlate well across scRNA-seq technologies, but not with single-cell protein measurements. However, the intensity-derived iBAQ abundance value of proteins is only a proxy for the absolute abundance of the individual protein and thus is limited in its capability to precisely rank proteins by their absolute abundance. Therefore, the lower correlation could be explained at least in part by this bias, which should at least be discussed in the text.

On page 13 and Extended data Fig. 5A, the authors claim that for protein expression measurements, coefficients of variation were very small across covered abundances, independent of the data completeness. I have to note that I cannot follow the argumentation via the poisson distribution. However, from looking at the data point distribution, a clear dependence of CV to abundance is visible for all three datasets including the protein data. Furthermore, data completeness in proteomics is usually highly dependent on intensity/abundance. Thus, I would expect that the lower abundant protein readouts have a higher fraction of missing values. This could be investigated with a scatterplot of proteins with their abundance vs. detection in # of cells.

On page 13, the authors describe the results found in Fig. 5E, Fig 5D and Extended data Fig. 5B, C. I think the claim "Interestingly, these proteins were distributed well across the covered dynamic range of the Proteome" disregards the clear trend towards higher intensities for the core proteome. And also the claim "we found the corresponding transcripts of the core proteome to be distributed across the full range of CVs in single-cell transcriptome data" disregards the trend towards overall lower CVs in RNA data of the core proteome. The authors should rephrase these descriptions.

Minor points

Putting together the manuscript appears rushed in places, with inconsistencies in cross-figure units (e.g. CV measurements in supp figs 2 & 4) which complicates the interpretation of fundamental conclusions made in the manuscript. Moreover, there are inconsistencies in the described methods, as the authors are presenting DIA-NN results for their main single cell data processing, but then continue on talking about Spectronaut tables in the methods section. Having seen the work being presented at several conferences over the past year, I am aware that the authors moved from using Spectronaut 14 (identifying ~1000 proteins per cell) to Spectronaut 15 (identifying ~1500 proteins per cell) and now, finally, to DIA-NN 1.8 (identifying ~2000 proteins per cell). As I cannot seem to find any other experimental optimizations that have been done to achieve such benefits, I am a bit puzzled how to interpret such vast improvements without a clear evaluation of this by the authors. E.g. how did the CV values of protein quantifications change with the different numbers of proteins identified, and how did the different search engines affect this? A comparison to a bulk proteomics HeLa dataset would also help clarify the quantitative accuracy and precision aspects of conducting proteomics at single cell level (as mentioned above).

In Fig. 5c the authors should clarify how they matched cells between datasets to calculate the correlation, since matching cell that are in different states across datasets would reduce correlation.

In Fig. 5b & c, the authors should clarify in the figure caption how many cells and proteins were used.

On Pg. 7, the authors claim that "while highlighting a substantial heterogeneity within each that would have been hidden in bulk sample analysis (Fig. 4C)". I'm not entirely sure how the boxplots are indicative of cellular heterogeneity.

In supp fig 2., authors present a median CV of 0.3 (assuming this means 30%?), but then in supp fig 4, suddenly seem to suggest CVs below 1. What units are used here, and I assume that 1 in this case means 10% CV? The units need to be standardized throughout and I wonder why there seems to be a discrepancy in the assessment of reproducibility.

On Pg 10, the authors state "suggesting that our single-cell protein analysis could benefit from imputation or tailored likelihood-based parameter estimation methods." Here it would be good with relevant references from the SCP field, such as Specht et al., 2021 or Schoof et al., 2021, who have already successfully shown the implementation and effects of imputation approaches in SCP data.

Reviewer #2:

This is a straightforward manuscript with a clear story line and written well. As there a lot of interest in single cell proteomics, this is a valuable contribution to this nascent field. Most of my comments are, therefore, minor. That said, this paper is an opportunity to guide readers as to what they may expect from single cell proteomics (and transcriptomics). Hence, I encourage the authors to provide more such guidance and mention the state of the art from other publications in this field more expliciteyl. This is important because there is practically no discussion section. Along the same lines, the authors should tone down the excitement about their own (good) data in places and rather give more space to outlining comparisons to other approaches and the general state of play. 2,000 proteins in a single cell is great, but a far cry from 10,000. I support publication of the work provided that the following points are addressed in a revised manuscript.

Specific points
Abstract: the claim that the technology is applicable to PTMs is not justified at present because it would require another 10x or so in sensitivity. It is not clear if/how that can be achieved with the current combination of sample preparation and mass spectrometer.
Main: the authors state that their technology delivers accurate quantification. That cannot be claimed. What they likely mean is quantitative precision. These two things are not the same.
Noise-reduced quantitative mass spectra:
a) it would be informative to see the dilution data in Fig 1C extended to levels of a single cell, better actually below in order to be able to gauge the sensitivity of the 'old'setup and that backs up the statement made about the estimate of needing 10x more sensitivity. The text could actually state more explicitely that the data in Figure 1 is 'old setup'
True single-cell proteome analysis:
a) how do the authors explain the identification of proteins in the 'zero' cell data (Fig 2B)? Is this carry-over from previous runs or protein contamination encountered durring sample preparation?
b) the authors should point out that MBR is not without errors, better quantify the extent of that error. This is important as MBR alone doubles protein IDs in the absence of MS2 data that would back up these IDs. The authors should also point out that the sensitivity of the mass spectrometer in MS2 is still limiting (as one would of course expect).
c) Figure 2C/D does not show quantification accuracy. It is precision at best. Please change the wording to something like 'reproducibility'.
d) The authors should point out in an appropriate place in the manuscript that FACS sorting itself could lead to changes in the proteome.
More than 10-fold sensitivity increase:
a) perhaps a matter of taste and really just a minor point, but seems a little odd to first introduce the improvements made on the mass spectrometer to then go back to sample preparation. A more logical flow might be to start with the sample prep and LC parts, then go to the mass spectrometer and then the IT component to see where the improvments come from.
b) the authors should comment on which proteins one would likley miss by lysis in organic solvent (or if that is not the case)
b) Figure 3C: I would not call 100 nl/min true nano-flow. It suffices to call it nano-flow. It also does not show single cell data. It would be much more compelling to place the results of actual single cell experiments here to support the frequent mentioning of 'true single cell'.
c) How was the DIA library constructed? The authors should point out the downside/risks of DIA as one cannot simply assume that errors in DIA data processing (which is already hard to estimate) would scale down to single cells. While the increase from 550 proteins to 3,900 proteins is impressive, what is that figure for single cells?
T-SCP dissects arrested cell-cycle states:
a) why was the DIA library confined to 4,000 proteins and how was that level chosen?
b) Have the authors checked by microscopy if G2 cells are about twice as large as cells in other stages of the cell cycle?
c) how was the 15% criterion chosen for filtering the single cell data?
c) could the authors comment on Figure 4D, specifically why PCA1 and 2 only capture relatively little of the variation in the data? Without the color code, it would be very diffiecult to distinguish the cell populations
d) Fig 4e: this model suggests that only G2-M can be distinguished from other cells. The AUCs for cells in S or G1 phase are below 0.5 which indicates that the model is worse than random for these cells. Please comment.
e) could the authors explain their intention behind showing the data in Fig 4G? Do you show this to 'validate' the results of the DIA software?
SC proteomes compared to transcriptomes:

a) please include the number of transcripts in the data set to give readers an idea of what part of the genome is covered at the transcript and protein level. This does not diminish the value of the single cell proteome data shown but provides guidance as to what people might expect to get when attempting such experiments
b) Fig 5B is not particularly telling (unless I missed it). It would have been great to see that comparison done on the cell cycle experiment. that would have been very very informative in terms of what the two different technologies can and cannot reveal. The technical comparisions shown are fine but I am not sure they drive a particularly strong point.
T-SCP reveals a stable core proteome
a) These findings are not surprising or are they? The authors should reference a few papers that looked at this at the bulk proteome/transcriptome level in the past and then use a narrative that says that what one sees on the bulk level is pretty much the same at the single cell level - at least for the very abundant proteins.
Outlook:
a) this is very short and the reference are a bit 'self-centered'. Therefore, the authors should provide more discussion in the respective result sections as outlined above.
b) This reviewer did not understand what the authors mean by: "Our workflow is also compatible with chemical multiplexing with the advantage that the booster channel causing reporter ion distortions could be omitted or reduced." Does this refer to the ion mobility dimension?

c)

Wednesday, December 22, 2021

Point by point response to reviewers

Reviewer #1

Summary

In their manuscript, Brunner et al. present a single-cell proteomics workflow that is facilitated by multiple significant advances in the field. The novel mass spectrometer presented (Bruker timsTOF SCP) shows a clear improvement in terms of sensitivity, which, in combination with the diaPASEF acquisition method and a novel implementation of the EvoSep One LC system, leads to great results in terms of proteome coverage of single cells. Especially when analyzed by sophisticated machine-learning based spectral interpretation engines (e.g. DIA-NN), the authors appear to reach unprecedented proteome depth in label-free single cells. Also in terms of cell throughput, the authors set a new standard (40 cells per day), although when put in the context of multiplexed approaches, their throughput arguably still falls short. Nevertheless, through a series of experiments focusing on manipulating the cell cycle in HeLa cells, the authors demonstrated a "core proteome" to exist, especially compared to transcriptome-level readouts which appear to be more stochastic. Overall, the authors demonstrate the power of teaming up with top-level industry partners, and provide an additional tool in the arsenal of newly established single cell proteomics by Mass Spectrometry (SCP) approaches.

We thank the reviewer for highlighting the benefit of our work the single-cell community. Also, we appreciate them acknowledging the intense collaboration between academia and industry that was necessary to achieve this.

General remarks

Overall, the key conclusions made throughout the manuscript seem generally sound, however, the authors on several occasions appear selective in placing their work in context of previous SCP work, which is somewhat problematic. For example in the opening paragraph, they end with claiming that "a technology that provides quantitatively accurate MS proteomics data from true single cell-derived signals (T-SCP) and answers biological questions is still outstanding". In my opinion, this does not do justice to efforts made on e.g. the NanoPots platform by Ryan Kelly, Ying Zhu and colleagues or alternative chip-systems by Catherine Wong et al, who have collectively absolutely pioneered taking MS based approaches to the single cell level, and have demonstrated their utility in a variety of biological questions. The main benefit of the approach presented here is the significantly improved cell throughput while keeping proteome depth high, and this is a significant development in its own right that deserves to be the main selling point in my opinion.

We agree with the reviewer that sample preparation technologies down to the level of single-cells has not been necessarily pioneered by us and we add citations of the mentioned authors wherever we could. Nanopots, initially developed for small sample amounts has indeed been adapted to the use of multiplexed single-cell analysis, which we acknowledge. Furthermore, accompanying citations for Catherine Wong's work have been added. We also made clear that the label-free single-cell analysis at scale and high throughput (for proteomics) is one of the main selling points of our manuscript.

The authors opt to apply a technology (single cell approaches) originally developed for detecting cellular heterogeneity to a homogeneous cell line, into which they introduce heterogeneity through a variety of cell cycle inhibitors. The exact biological impact of this particular investigation, beyond comparing it to transcriptome level readouts, still somewhat eludes me, and I am not convinced that in terms of cell cycle regulation at the protein level, that any new biological insights were generated compared to previous bulk-cell level investigations. Perhaps more cells would be needed to truly detect distinct cell states within their enriched populations, but this is not really discussed in the manuscript. If the authors' main intention was to demonstrate the ability of their SCP workflow to read-out different cell states, then this was successful.

We thank the reviewer for pointing this out. Indeed, our main interest was the establishment of a highly quantitative and robust label-free single-cell proteomics workflow that is scalable. We used the analysis of single-cells pushed into different cell cycle stages to recapitulate known cell cycle markers as highlighted in the manuscript and also to highlight variability on the single-cell level within the treatment pools.

The observations about the core proteome, especially when compared to the transcriptome are very interesting and provide some novel insights into molecular cell biology with single cell resolution. It was interesting that they spanned the covered dynamic range of the proteome, and that the corresponding CVs at the transcriptome level did not suggest such a high level of stability. This work as a whole is a clear example of how MS-based proteomics provides a complementary view to RNAseq, and now also at the single cell level. As pointed out by the authors, these core proteins could represent appropriate loading controls on e.g. western blot or PRM analysis and was a neat spin-off conclusion to their data exploration efforts.

Highlighting that the transcriptome is rather stochastic as compared to the proteome, which is rather stable, is indeed one of our main observations representing fundamental differences on both modalities at the cellular level. It also highlights that much of the proteome needs to remain quantitatively stable to maintain cellular homeostasis, while the transcriptome is more volatile overall.

Major points

As described above, the authors present a convincing experimental workflow which lifts label-free quantification of protein levels using MS to the next level. However, I find that the manuscript falls short in the evaluation of the quantitative accuracy of the up to 2000 proteins measured in some of the single-cells. Furthermore, I have multiple concerns about the comparison of the measured proteome to the measured transcriptome as detailed below:
On page 4 and figure 2C & D, the authors claim high quantitative accuracy by comparing abundance values of proteins between measurements. However, this analysis only evaluates the quantitative precision across the intensity range and reveals that precision is highest at high abundance and decreases towards lower abundances. The authors should evaluate the quantitative accuracy of their readout by comparing measured fold-changes on single-cell level to fold-changes measured to bulk data (i.e. approximation of the ground truth); this analysis has been performed extensively for SCoPE-MS experiments around the world. Alternatively, quantitative accuracy could be evaluated by providing ground-truth fold-changes in the experimental design e.g. by spiking in known peptide amounts/ratios. I hypothesize that quantitative accuracy is highly dependent on signal intensity, and thus, it would important to investigate how many of the up to 2000 proteins provide a reliable quantitative readout and can be used for biological interrogation. The authors repeat that mis-interpretation on page 8 and Extended data Fig. 2D, by presenting the coefficient of variation (CV), which is a measure of precision, and not quantitative accuracy, as claimed in the text.

We acknowledge the reviewer's point that CVs measure precision rather than the accuracy. In the revised manuscript we are careful to point this out. However, label-free quantification (in contrast to TMT-based quantification, for instance), has little or no tendency to systematically skew quantitative ratios. This has been shown throughout the proteomics literature, and in particular in few protein or whole proteome spike-in experiments (now cited in the revised manuscript) (Cheung et al., 2021). Furthermore, our measurements of increasing cell numbers (one to eight) show exactly the expected patterns and no bias is apparent. Again, this is in contrast to TMT-based measurements, where the signal for eight cells would not be eight-fold higher than the signal for a single cell.
We completely agree with the reviewer, that quantitative accuracy and precision depends on the signal intensity of each protein and peptide. We now document this in a new Suppl. Figure 2F, which shows the dependence of the CV as a function of protein signal in addition to the total CV count across the data set (S2E):

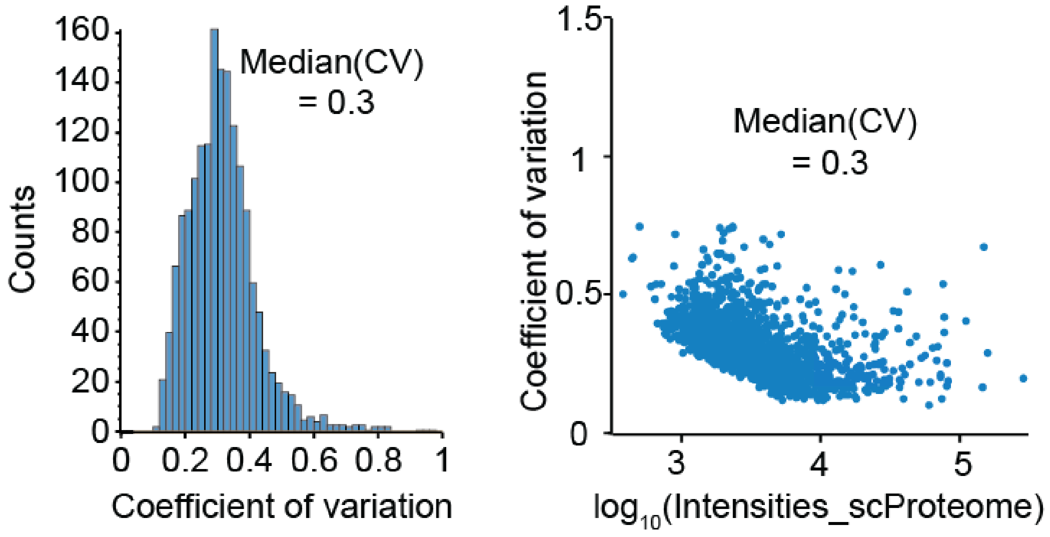

Regarding the request for additional measurements (see also reviewer 3), we agreed with the editor that this would be beyond the scope of timely publication of this paper.

On page 10 and Extended data Fig. 4A, the authors claim that cells have higher correlation on average than in the droplet-based method and similar correlation to the SMART-Seq2 method. However, I assume that this analysis was done with all genes measured [T-SCP (Cells x Proteins: 424 x 2,480), SMARTseq2 (Cells x Genes: 720 x 24,990), and Drop-seq (Cells x Genes: 5,383 x 41,161)]. Thus, the trend of the strength of correlation follows the amount of genes measured, and therefore could simply reflect the decreased number of reads per gene, and thus higher measurement noise, if more genes are measured. The authors should compare the correlation of the cells using the same genes/proteins across the datasets or take the top 2,480 genes in the rna-seq datasets to compare to the 2,480 proteins.

We thank the reviewer for raising this important point. Indeed, we compared the set of proteins in our single-cell proteomics measurements filtered to at least 600 protein identifications and 15% data completeness to the RNA-seq also filtered for at least 600 gene identifications and 15% completeness, then scaled to the average cell size of each particular data set. In our view, this ensures the fairest comparison of the current status of the two most prominent RNA-seq technologies to the current status of our single-cell proteomics workflow. As reported by e.g. Svensson et al. (referenced in our paper)(Svensson, 2020; Svensson et al., 2017), scRNA-seq has reached a limit in sensitivity and quantitative accuracy, thus RNA-seq data are not ‘zero inflated’ but this is an inherent feature of the sc transcriptome.
Still, we performed the presented analysis on shared genes only across the proteome and transcriptome (S4A (Right)) keeping in mind that the represented MS-based data will inevitably represent a natural higher quantitative variability at the lower abundance level since we are fully sensitivity limited, while the scRNAseq technologies are not:

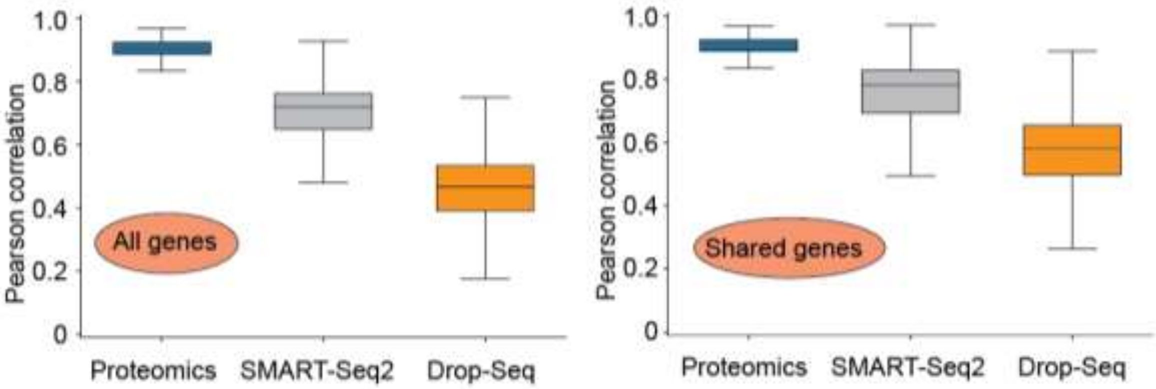

Similarly, on page 10 and Fig. 5A, the authors show decreased data completeness for the sc-RNAseq methods. However, these methods also measured many more genes. For a fair comparison, the authors should compare the data completeness of the same genes/proteins across the datasets or take the top 2,480 genes in the rna-seq dataset to compare to the 2,480 proteins. Comparing only these subsets could also change the observation of bimodal gene completeness frequency distribution in sc-RNAseq in Extended data Fig. 4b.

We performed the analysis the reviewer asks for keeping in mind the argumentation above with regards to the juvenile label-free LC-MS based single-cell proteomics technology and the very mature scRNAseq technologies:

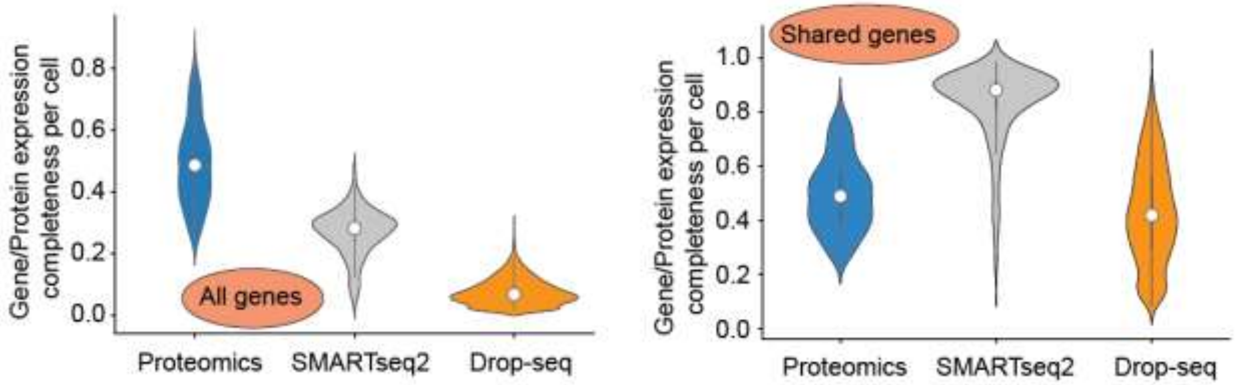

The analysis on shared genes mainly reveals that SMARTseq2 seems to show higher correlations than the proteome for this data set, while Drop-seq is far off. This can be explained by the fact that the SMARTseq2 data is not UMI-controlled and is therefore prone for saturating sequencing – especially for highly abundant transcripts, which consequently results in high correlations, which does not reflect the biological truth. This is in stark contrast for Drop-seq, which is UMI-controlled and results in a controlled gene completeness distribution. In our opinion, the analysis on shared genes rather reflects a clear technical limitation of the SMARTseq2 data set instead of supporting our initial aim, which was the comparison of the "is-state" and its gene/protein expression completeness per cell.

On page 10/11 and Fig. 5B, the authors show that protein measurements separate strongly from RNA in a principal component analysis. I'm a bit concerned that this observation could be biased by including missing values in the comparison, as the underlying reason for missing values could be technology dependent. I suggest to repeat the analysis with a smaller subset of cells and overlapping genes that are completely quantified in all methods. Alternatively, missing values could be imputed, instead of replacing them with 0. Furthermore, I would suggest to scale the data to zero mean and standard deviation 1 to remove potential bias of different dynamic ranges of the different data types. Finally, if the results turn out to be similar, I would suggest to investigate which genes drive the separation of the protein data from the RNA data in principal component 2, as this would be of great biological interest.

We thank the reviewer for this careful observation. Indeed, we were heavily thinking about how to perform a fair global analysis. The main issue in integrating the data is first, the data distribution and second, the different ways of preparing the data for downstream analysis in the proteomics and single-cell RNAseq community. The overall data distribution looks like this without taking into account any zeros (S4B now):

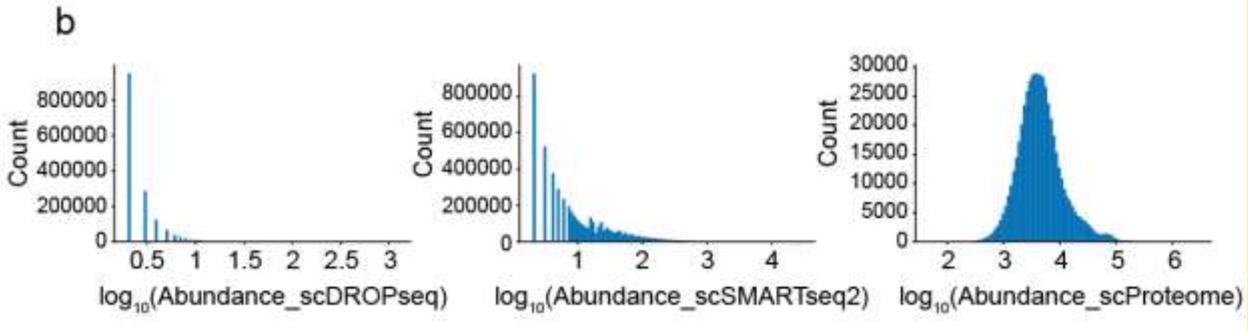

Due to the fact that single-cell RNAseq data count data that are dominated by count noise and zero counts are not necessarily the result of a technical bias as shown by Svensson et al ((Svensson, 2020; Svensson et al., 2017) since sensitivity limits have been shown to be reached, we decided to take zero values as the biological truth in this comparison. In contrast, proteomics data follow a normal distrubtion, are not zero inflated and absence of quantitative values does not equal the absence of the protein within the analyzed sample, which is why we did not zero-impute the missing values within the data set, but scale all data to the mean cell size of each technology. Our goal was to show that the nature of the proteome and the transcription is inherently different, which is also represented by the way of how both communities process the data.

We thought about the reviewer's suggestion of scaling the data for comparative reasons, but decided that standardizing the data would remove way too much of its data distribution. Nevertheless, we agree with the reviewer that our way of analyzing the data might not be ideal to make the point for the biological difference between the transcriptome and proteome. To be as independent as possible of the experimental design and technology and preserving data distribution, we decided to calculate the coefficient of variation for each gene within each data set for all shared genes, followed by correlation analysis across the technologies. `

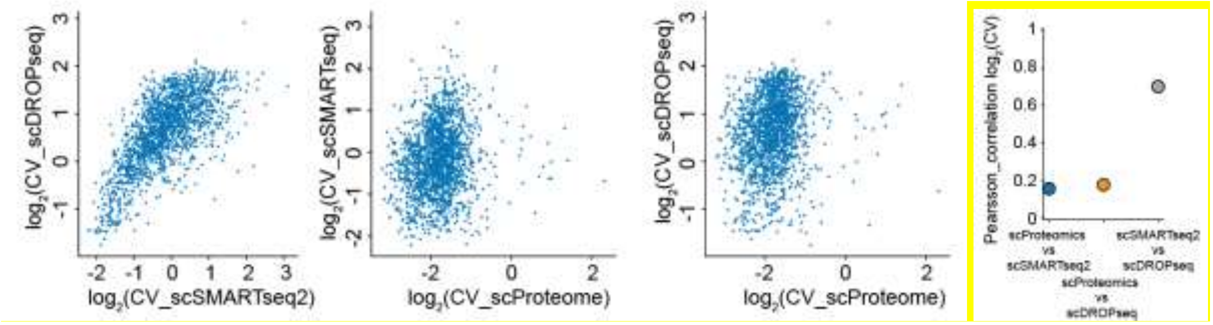

Again, this analysis highlights that the transcript variability is low (Pearsson correlation is high) within the scRNAseq technologies, but high between scProteomics and the scRNAseq technologies (Pearsson correlations is low). We added this analysis as S4C, S4D.

On page 11 and Fig. 5c, the authors claim that single-cell transcript expression levels correlate well across scRNA-seq technologies, but not with single-cell protein measurements. However, the intensity-derived iBAQ abundance value of proteins is only a proxy for the absolute abundance of the individual protein and thus is limited in its capability to precisely rank proteins by their absolute abundance. Therefore, the lower correlation could be explained at least in part by this bias, which should at least be discussed in the text.

For the reasons that the reviewer mentions, we do not use iBAQ data in our quantitative analysis but rather summed intensities. This is now pointed out more clearly in the revised manuscript.

On page 13 and Extended data Fig. 5A, the authors claim that for protein expression measurements, coefficients of variation were very small across covered abundances, independent of the data completeness. I have to note that I cannot follow the argumentation via the Poisson distribution. However, from looking at the data point distribution, a clear dependence of CV to abundance is visible for all three datasets including the protein data. Furthermore, data completeness in proteomics is usually highly dependent on intensity/abundance. Thus, I would expect that the lower abundant protein readouts have a higher fraction of missing values. This could be investigated with a scatterplot of proteins with their abundance vs. detection in # of cells.

What we wanted to bring across is that the sc transcriptome is dominated by shot noise, which has a Poisson distribution, because many of the transcripts are expressed at lower than one copy per cell on average. This means that a given single cell can have zero, one or two transcripts of many genes, leading to a Poisson distribution when summing up many single cell measurements. For genes with higher expression values, there will always be transcripts present in each single cell and then the expression distribution of those genes is not shot noise dominated. For proteins, the CVs depend on the measurement, whereas there are always sufficient copied in each cell to ensure that their expression levels are not

biologically shot noise limited. We have made this central point - which is also the underlying reason for the 'zero inflation' mentioned above – more clearly in the revised manuscript. We also describe more clearly our completeness plot – log2 mean of abundance vs. percentage completeness in single cells (EV 2A).

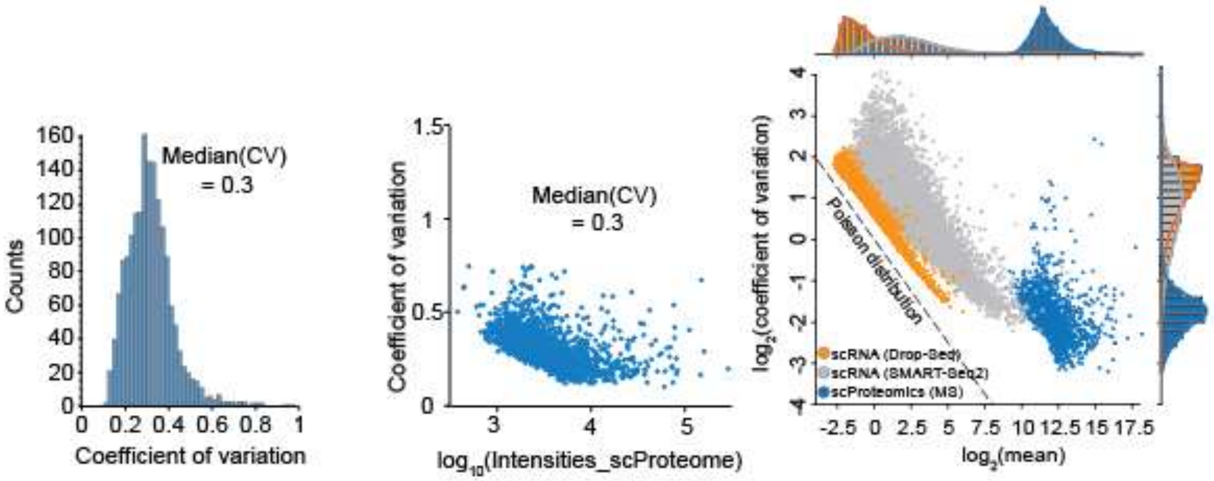

On page 13, the authors describe the results found in Fig. 5E, Fig 5D and Extended data Fig. 5B, C. I think the claim "Interestingly, these proteins were distributed well across the covered dynamic range of the Proteome" disregards the clear trend towards higher intensities for the core proteome. And also the claim "we found the corresponding transcripts of the core proteome to be distributed across the full range of CVs in single-cell transcriptome data" disregards the trend towards overall lower CVs in RNA data of the core proteome. The authors should rephrase these descriptions.

We rephrased these descriptions in the mentioned text parts. We emphasize that the core proteome does depend on current technology but that it represents a genuine underlying phenomenon.

Minor points

Putting together the manuscript appears rushed in places, with inconsistencies in cross-figure units (e.g. CV measurements in supp figs 2 & 4) which complicates the interpretation of fundamental conclusions made in the manuscript. Moreover, there are inconsistencies in the described methods, as the authors are presenting DIA-NN results for their main single cell data processing, but then continue on talking about Spectronaut tables in the methods section. Having seen the work being presented at several conferences over the past year, I am aware that the authors moved from using Spectronaut 14 (identifying ~1000 proteins per cell) to Spectronaut 15 (identifying ~1500 proteins per cell) and now, finally, to DIA-NN 1.8 (identifying ~2000 proteins per cell). As I cannot seem to find any other experimental optimizations that

have been done to achieve such benefits, I am a bit puzzled how to interpret such vast improvements without a clear evaluation of this by the authors. E.g. how did the CV values of protein quantifications change with the different numbers of proteins identified, and how did the different search engines affect this? A comparison to a bulk proteomics HeLa dataset would also help clarify the quantitative accuracy and precision aspects of conducting proteomics at single cell level (as mentioned above).

Indeed, we did not perform any experimental optimizations and the improvements in the numbers are a result of rapid improvements in Spectronaut and MS-Fragger combined with DIA-NN for TIMS-TOF data. In the initial Bioarchive paper, the MS-Fragger was not yet compatible with DIA-NN and DIA-NN for TIMS-TOF was only available end of June this year. Spectronaut then mostly caught up with these developments about one month later. These are major developments that indeed utilize the complex diaPASEF TIMS-TOF data much better, explaining the large improvement in sc protein numbers.
Specifically, this included the release of Spectronaut 15 and DIANN 1.8 for the analysis of timsTOF data. The initial analysis presented at conferences was indeed performed with Spectronaut 14. Spectronaut 15 was updated with additional machine learning algorithms that benefited signal deconvolution in DIA data in general. DIA-NN itself has been shown beneficial especially for the analysis of short gradient and also limited sample amounts (DIANN, DIANN COVID). For instance, the DIA-NN authors reanalyzed our original diaPASEF data from the Nature Methods paper, dramatically improving identification number, especially in short gradients; in keeping what we see here for single cell data (ref DIANN low sample amounts). According to the authors, this benefit results from the advanced use of deep learning algorithms for signal deconvolution and for diaPASEF data specifically from the additional ion mobility resolution that makes full use of the correlation between ion mobility and mz on MS2-level for fragment signal pattern identification and its matching to the precursor level. That said, our main focus in the current manuscript was not to present computational advances and a benchmarking of DIANN versus Spectronaut, but to highlight our scalable and robust MS-based single-cell proteomics workflow. Prompted by the reviewer's comments, we now comment on the improvements in software in the revised manuscript. Furthermore, because of its somewhat better performance and to present a unified analysis, we now consistently analyze all data with DIA-NN.

In Fig. 5c the authors should clarify how they matched cells between datasets to calculate the correlation, since matching cell that are in different states across datasets would reduce correlation. The description of the correlation analysis can be found in the methods section: "For correlation analysis, the RNA abundance entries were linearly scaled to sum to the mean cell size of the respective dataset per cell (231,281.56 for SMART-Seq2 and 7,808.12 for Drop-Seq) followed by log(x+1)-transformation of all abundance entries. Correlation values between the expressions of two cells were computed as the Pearson

correlation on the 1,672 genes that were shared in all 3 datasets. Entries of missing protein abundance values were excluded from the specific computation."

In Fig. 5b & c, the authors should clarify in the figure caption how many cells and proteins were used.
We added the number of cells and proteins that were used for the analysis.

On Pg. 7, the authors claim that "while highlighting a substantial heterogeneity within each that would have been hidden in bulk sample analysis (Fig. 4C)". I'm not entirely sure how the boxplots are indicative of cellular heterogeneity.
We are sorry for the confusion here. We wanted to highlight a substantial heterogeneity within each of the drug treatments that would have otherwise been hidden in bulk analysis. This is now clarified in the revised manuscript.

In supp fig 2., authors present a median CV of 0.3 (assuming this means 30%?), but then in supp fig 4, suddenly seem to suggest CVs below 1. What units are used here, and I assume that 1 in this case means 10% CV? The units need to be standardized throughout and I wonder why there seems to be a discrepancy in the assessment of reproducibility.
We thank the reviewer for this observation. Indeed, we represent CVs as percentage (0.3 = 30%) throughout the manuscript. In S2 (now EV2A and B) we represent CVs as calculated. In S4, now S6, we log2-transformed the CVs when comparing transcriptome to proteome data.

On Pg 10, the authors state "suggesting that our single-cell protein analysis could benefit from imputation or tailored likelihood-based parameter estimation methods." Here it would be good with relevant references from the SCP field, such as Specht et al., 2021 or Schoof et al., 2021, who have already successfully shown the implementation and effects of imputation approaches in SCP data.
We added accompanying references for possible imputation strategies.

Reviewer #2

This is a straightforward manuscript with a clear story line and written well. As there a lot of interest in single cell proteomics, this is a valuable contribution to this nascent field. Most of my comments are, therefore, minor. That said, this paper is an opportunity to guide readers as to what they may expect from single cell proteomics (and transcriptomics). Hence, I encourage the authors to provide more such guidance and mention the state of the art from other publications in this field more explicitely. This is important because there is practically no discussion section. Along the same lines, the authors should tone down the excitement about their own (good) data in places and rather give more space to outlining comparisons to

other approaches and the general state of play. 2,000 proteins in a single cell is great, but a far cry from 10,000. I support publication of the work provided that the following points are addressed in a revised manuscript.

We thank the reviewer for the positive feedback. Indeed, up to 2,000 protein identifications per cell only represent a fraction of the total proteome. However, it is not very far from what sc transcriptomics measures of protein coding genes per cell and single run proteomics with unlimited input material does not achieve 10,000 proteins as a rule either. Nevertheless, we toned down our excitement wherever possible and incorporated all points of discussion.

Specific points

Abstract: the claim that the technology is applicable to PTMs is not justified at present because it would require another 10x or so in sensitivity. It is not clear if/how that can be achieved with the current combination of sample preparation and mass spectrometer.

The statement the reviewer alludes to is in the context of what the ultra-high sensitivity workflow could be used for. We now clarified this statement by highlighting that the analysis of PTMs from very limited sample amounts or cell-type resolved cell-pools comprising only hundreds of single-cells is now in reach.

Main: the authors state that their technology delivers accurate quantification. That cannot be claimed. What they likely mean is quantitative precision. These two things are not the same.

We thank the reviewer for raising this point, which was also highlighted by reviewer 1. Please see answers to that reviewer above.

Noise-reduced quantitative mass spectra:

a) it would be informative to see the dilution data in Fig 1C extended to levels of a single cell, better actually below in order to be able to gauge the sensitivity of the 'old' setup and that backs up the statement made about the estimate of needing 10x more sensitivity. The text could actually state more explicitly that the data in Figure 1 is 'old setup'

We edited the text and state more explicitly that the old setup was used in figure 1. Diluting the sample input down to the level of single-cells on the old instrument did not consistently yield protein identifications that were distinct from the blank injections, although some experiments resulted in up to 300 proteins. Identifying 800 proteins from less than 1ng of input material not taking into account losses from sample preparation, and keeping in mind that a single cell contains about 150pg of protein in total, we set ourselves

the challenge to increase the sensitivity of our LC-MS setup at least by 10-fold to be able to identify a reasonable number of proteins.

True single-cell proteome analysis:

a) how do the authors explain the identification of proteins in the 'zero' cell data (Fig 2B)? Is this carry-over from previous runs or protein contamination encountered during sample preparation?

We cannot be entirely sure, where these identifications come from, but most likely, as the reviewer suggests, they are due to a combination of carry-over at these extremely low input amounts and protein contaminations. We now provide a rank plot of these proteins as extended data figure (EV1A), which indicates that all proteins without MBR stem from high abundant proteins, which are most likely carryover from previous runs that were loaded with peptides. Indeed, MBR results in a lot more protein identifications as pointed out and also mentioned during the manuscript, including citations that try to quantify the MBR error. Still, this does not affect our further robust analysis of the single-cell proteomes, since we switched to a globally controlled data independent acquisition method for this.

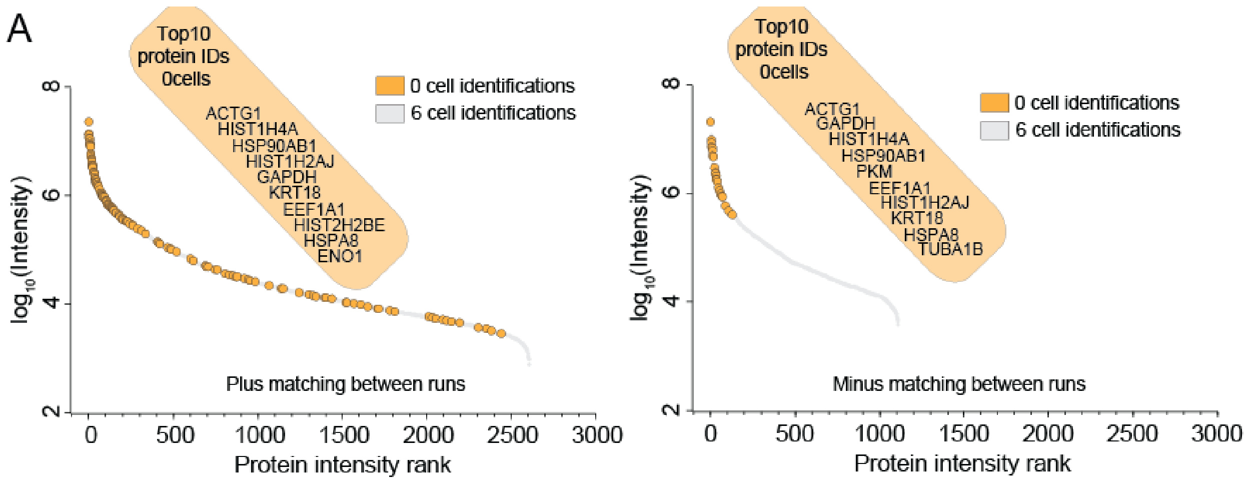

b) the authors should point out that MBR is not without errors, better quantify the extent of that error. This is important as MBR alone doubles protein IDs in the absence of MS2 data that would back up these IDs. The authors should also point out that the sensitivity of the mass spectrometer in MS2 is still limiting (as one would of course expect).

We agree with the reviewer and incorporated the suggestion in the revised manuscript at multiple points.

c) Figure 2C/D does not show quantification accuracy. It is precision at best. Please change the wording to something like 'reproducibility'.

Thank you, we fixed this now.

d) The authors should point out in an appropriate place in the manuscript that FACS sorting itself could lead to changes in the proteome

We incorporated this suggestion in the methods part.

More than 10-fold sensitivity increase:
a) perhaps a matter of taste and really just a minor point, but seems a little odd to first introduce the improvements made on the mass spectrometer to then go back to sample preparation. A more logical flow might be to start with the sample prep and LC parts, then go to the mass spectrometer and then the IT component to see where the improvments come from.

In reality the improvements in the hardware were mainly made at Bruker in Bremen, whereas the Munich lab worked in parallel on other aspects of the sc workflow. Furthermore, both were iterative processes. Indeed, we were debating about the flow of information, too, but decided that the presented way is the one that does most justice to how the workflow developed.

b) the authors should comment on which proteins one would likely miss by lysis in organic solvent (or if that is not the case)

We agree on this and added textual changes, especially since every sample preparation with its individual protein solubilization agents will inevitably results in slight differences in protein recovery. Still, we extensively compared ACN-based protocols to e.g. SDC-based protocols and did not observe any specific difference in sample protein recovery and consequently adopted this protocol for the deep visual proteomics work in our laboratory that aims at the analysis of cell-type resolved cell populations from FFPE tissue.

c) Figure 3C: I would not call 100 nl/min true nano-flow. It suffices to call it nano-flow. It also does not show single cell data. It would be much more compelling to place the results of actual single cell experiments here to support the frequent mentioning of 'true single cell'.

The difference here is most striking with respect to the original 1 ul/min flow of the Evosep. However, we agree and changed the wording accordingly.

d) How was the DIA library constructed? The authors should point out the downside/risks of DIA as one cannot simply assume that errors in DIA data processing (which is already hard to estimate) would scale

down to single cells. While the increase from 550 proteins to 3,900 proteins is impressive, what is that figure for single cells?

We constructed the DIA library from six single run analyses of 50 ng of pooled cells without regards to the cell cycle state. This is the most straightforward way to construct a library and we deliberately did not make the library too large, which would make FDR control difficult. In our opinion, this was the most conservative way to approach matching in diaPASEF at extremely low sample amounts.
During the course of the project, both the standard Bruker timsTOF instrument and the 'single cell' timsTOF instrument were continuously developed further. Thus, while we have documented the increase the reviewer mentions above, it is not possible any more to restore the original performance of the old instrument. However, our data is consistent and we would expect a similar factor.

T-SCP dissects arrested cell-cycle states:
a) why was the DIA library confined to 4,000 proteins and how was that level chosen?

As mentioned just above, our goal was not to create the deepest library possible for the analysis of single-cell derived DIA files, but to keep the library consisting of a six single-shot runs rather small since we did not expect the presence of proteins exceeding this number within our samples. Furthermore, as the reviewer pointed out before, the proteomics community suspects an inflation of false-positive identifications with increasing spectral library size. To counteract this, we kept the library on purpose at rather low levels of saturated single-shot analyses.

b) Have the authors checked by microscopy if G2 cells are about twice as large as cells in other stages of the cell cycle?
No, we did not, but it is a well-established concept in cell biology that cells grow during cell cycle progression. In response to the reviewer's comment, we now do not mention a doubling (which will anyway depend on the cell type).

c) how was the 15% criterion chosen for filtering the single cell data?
The 15% filter criterion was chosen with the goal to keep proteins within the data matrix that have been quantified in at least 60 observations across the experiment.

d) could the authors comment on Figure 4D, specifically why PCA1 and 2 only capture relatively little of the variation in the data? Without the color code, it would be very diffiecult to distinguish the cell populations
The cell cycle represents a continuum of changes, even when drug perturbed and arrested, which is why the cell populations overall are relatively similar. Conversely, within the same population of drug perturbed cells large variations are present, which is well known for experiments involving cell cycle arrest with

chemical inhibitors, followed by release. Note that these would have been averaged out in bulk experiments but are resolved here at the single cell level.

e) Fig 4e: this model suggests that only G2-M can be distinguished from other cells. The AUCs for cells in S or G1 phase are below 0.5 which indicates that the model is worse than random for these cells. Please comment.

For this analysis, we tested whether we can distinguish the set of G2-M cells from the set of G1-S cells using three different scores separately; each based on a set of known markers for G1, S and G2-M phase, respectively. Each score was used to generate one ROC curve separating G2-M cells (as positives) against G1-S cells (as negatives). This resulted in a 'better than random' ROC curve for the G2-M score that assigned high values to G2-M cells and a 'worse than random' ROC curve for the S and G1 phase scores that assigned systematic lower values for G2-M cells and higher values for the G1-S cells. We realized that this presentation of the results is a bit confusing and changed the plot to show the ROC curves with the targeted set of cells as positive target.

f) could the authors explain their intention behind showing the data in Fig 4G? Do you show this to 'validate' the results of the DIA software?

Figure 4G is in our opinion one of the highlights of the paper. It is not meant to validate the DIA software but rather to show at be fundamental data level that we are actually generating clearly interpretable data even at the single cell level. This is important because it could have been that the sc data was uninterpretable when visualizing individual spectra and that the data come out of the noise using advanced machine learning. However, this is not the case even at the fragment spectral intensity level and we can robustly quantify fold changes of proteins from single-cells when injecting cells one at a time into the mass spectrometer. This point is mainly aimed at experts in the MS field but it is very important in our opinion.

SC proteomes compared to transcriptomes:

a) please include the number of transcripts in the data set to give readers an idea of what part of the genome is covered at the transcript and protein level. This does not diminish the value of the single cell proteome data shown but provides guidance as to what people might expect to get when attempting such experiments.

We added the reviewer's suggestions to all figure descriptions.

b) Fig 5B is not particularly telling (unless I missed it). It would have been great to see that comparison done on the cell cycle experiment. that would have been very very informative in terms of what the two

different technologies can and cannot reveal. The technical comparisions shown are fine but I am not sure they drive a particularly strong point.

Figure 5B includes all data presented within the manuscript including the cell cycle data. In our opinion, this analysis highlights again the complementarity of the proteome and transcriptome level, especially since both single-cell transcriptome technologies heavily segregate from the proteomics measurements but not within the two transcriptomics workflows. We have added a sentence highlighting the purpose of the figure and expanded this part in response to reviewer 1’s question.

T-SCP reveals a stable core proteome

a) These findings are not surprising or are they? The authors should reference a few papers that looked at this at the bulk proteome/transcriptome level in the past and then use a narrative that says that what one sees on the bulk level is pretty much the same at the single cell level - at least for the very abundant proteins.

The reviewer is correct in stating that there are many papers reporting relatively small correlations between proteomics and transcriptomics at the bulk level (often 0.3 to 0.6). However, the point here is entirely different and is a major take home message from our work. We show that the transcriptome is stochastic because only very few copies of transcripts are present for most genes (leading to shot noise) whereas protein copy numbers are much higher and not subject to shot noise. Please see answer to reviewer 1 on page xx as well. We edited the text accordingly and also now point out that bulk correlations of transcriptome and proteome have frequently been found to have moderate correlations.

Outlook:

a) this is very short and the reference are a bit 'self-centered'. Therefore, the authors should provide more discussion in the respective result sections as outlined above.

We extended discussions in the appropriate result sections as suggested by the reviewer above.

b) This reviewer did not understand what the authors mean by: "Our workflow is also compatible with chemical multiplexing with the advantage that the booster channel causing reporter ion distortions could be omitted or reduced." Does this refer to the ion mobility dimension?

The main appeal of our single-cell proteomics workflow is that we actually reached the LC-MS sensitivity necessary for the analysis of single-cells. Every peptide signal that we identify stems from proteins that have been processed and digested from the initial single-cell. This is very different from the current TMT approach a booster channel is heavily populated by bulk peptides from a pool of cells, whereas single-cells are distributed across free channels within the same plex. This means that the MS1- and MS2-level signals

are heavily boosted by the bulk cell pool and ease the challenge of peptides identification by an order of magnitude or more. The fact that quantification comes the signal of single cells in the reporter channels can lead to extensive issues as discussed by many recent papers, for example Rose & Kuster, who conclude that the booster channel should not exceed 10 cells. This is the reason why we do not focus on the multiplexing approach.

That said, there is nothing to prevent scientists to employ a TMT-based multiplexing strategy with our workflow, leading to an increase in throughput (or at least a decrease in MS-time). The reviewer is correct that ion mobility would decrease 'ratio compression' effects inherent to TMT (Ogata Kosuke, Analytical Chemistry, 2020) and employing complementary TMT (Wühr Martin, Analytical Chemistry, 2012) or EASI-tag (Virreira-Winter Sebastian, Nature Methods, 2018) should eliminate it altogether. We make this point more clearly in the revised manuscript.

Reviewer #3

Summary

In this paper, a new workflow for single cell proteomics is described. Several parameters have been optimised to increase sensitivity and reproducibility, which includes optimisation of the mass spectrometer (introduction of the TIMS-TOF SCP (Bruker), nanoFlow LC-gradient, and data independent acquisition method. They applied their workflow to analyse the cell cycle and were able differentiate the different cell stages and identify new markers. Comparison to two single-cell transcriptomic methods revealed that the proteome did not correlate well with the transcriptome underlining the need for single-cell proteomics analysis. Moreover, the coefficient of variation was considerably lower for the proteomics workflow than that of both transcriptomics methods.

The manuscript describes the first pipeline for quantitative label free single cell proteomics and demonstrates its application to a well known biological system (cell cycle) using a commonly used human cell line (HeLa cells). So far the state-of-the-art approach for single cell proteomics is a TMT-based approach, which uses a booster channel/ However, this method presents intrinsic limitations, such as amount of labels/difficulties in interbatch comparisons (Brenes, A. et al MCP 2019, https://doi.org/10.1074/mcp.RA119.001472) and inaccuracy with increasing booster to single cell ratios (Cheung, TK et al, 2021, 10.1038/s41592-020-01002-5). The only other publication where single cell analysis has been performed without labels (also mentioned by authors) focused on sample prep and in fact only mentioned identifications but not quantifications of proteins. (Ling, Y. et al Anal. Cem. 2021, https://dx.doi.org/10.1021/acs.analchem.0c04240)

General remarks

The method described and the conclusion obtained from the comparison of proteome and transcriptome analyses look very convincing, although a couple of additional experiments would greatly strengthen the manuscript. In particular, the analysis of a different cell type and the comparison to the current state-of-the-art approach (TMT-based approach with booster channel) would greatly increase the impact of the manuscript and provide an important reference for the field. Additionally, the authors should make an effort to clearly describe in detail all the sample processing procedures, acquisition methods and data analysis pipelines, so that this new exciting method can be easily implemented in other laboratories.

We thank the reviewer for this positive evaluation and their judgement that this workflow is novel for the applications in single-cell proteomics since it is label-free and scalable.

Major points

The study seems solid, but it would be extremely important to compare these results directly with some other single cell analysis studies. The authors should use their workflow to analyse a different cell line (Jurkat and U-937, both commercially available) that has been previously analysed with single proteomics approaches based on booster channel, e.g., Specht, H et al. 2021, Genome Biology, https://doi.org/10.1186/s13059-021-02267-5, Budnik et al. 2019, https://doi.org/10.1186/s13059-018-1547-5 . The data from previous studies are publicly available and therefore they could be directly compared to the results obtained using the label free approach described here. With the throughput stated by the authors (40 cells per day), an analysis of a sufficient number of cells from a new cell line should not take more than a week of instrument time. Comparative analyses should include drop-out rate, CV at peptide and protein level, proteome coverage, data completeness, etc...

We agree that an assessment between the TMT- and label-free based workflows would be of major interest and interesting. Still, we think that a back-to-back comparison involving the same cell line and scale of the experiment would drastically shift the focus of this manuscript, which is the development of a robust and scalable label-free single-cell proteomics workflow followed by a comparison to the single-cell transcriptome. As evidenced by comments from all three reviewers our manuscript structure enabled us to highlight a fundamental biological difference at the single-cell level already covered a lot of ground and was hard to follow in parts. Adding a full blown analytical comparison to the TMT approach which has its own challenges would clearly exceed the scope of this report.
Furthermore, it was not our intention to ultimately make a recommendation for either of the two workflows, which would inevitably result from this. Therefore, we would like to refrain from this suggestion, which could still be topic of follow-up publications by us or others. Furthermore, we agree with the reviewer that the

analysis of a second cell-type to verify the behavior of the single-cell proteome versus transcriptome would be beneficial to further strengthen our observation, but we are convinced that this phenomenon is generalizable due to comparisons on bulk level that show the same trend and also much literally on single-cell transcriptomics that highlights its overall stochasticity. These points have also been discussed and agree to with the editor.

The authors should more openly report the potential limitations of their method. For example, what is the typical dropout rate, i.e., what fraction of cells where measured but did not yield a sufficient number of identifications and therefore need to be excluded?

We agree and comment more prominently on the exclusion of cells for downstream analysis due to our filtering steps.

The authors introduce a new instrument TIMS-TOF SCP, but the comparison to the previous generation instrument is rather superficial and limited to increased raw signal and summed peptide intensities (Fig.2A). Since the previous generation instrument is quite popular, it would be very important information for labs that already own an instrument to know what is the gain of the newly introduced instrument for single cell analysis. The author should include some comparison of single cell analysis performed on the previous generation instrument coupled to the same LC-system used with the TIMS-TOF SCP.

Thank you for point this out. Please see answers to reviewer 1 and reviewer 2 above.

Given that the major novelty of the manuscript is the introduction of a new workflow and that, as the author states, it can be envisaged that the method will become widely used by the community, it is extremely important that the authors provide sufficient details for the approach to be easily implemented by other labs. Specifically: A new EvoSep gradient has been mentioned, but it has not been specified what the gradient is or whether it is available. Please clarify.

We agree and are happy to report that the instrument described here has in the meantime become commercially available from Bruker as the TIMS-TOF SCP. Furthermore, Evosep has since released the low flow methods developed here under the name of 'Whisper'. Prompted by the reviewer we have added to the information we already provided to MSB within the methods section and added also crucial hardware parts as a table and ordering numbers. We now describe the methods in more detail, especially the EvoSep part and the EvoTip loading procedure since this was crucial in our hands. The new EvoSep Whisper gradient is now 28min long and accessible to the EvoSep community vial the official EvoSep homepage. To gain the 7 min reduction, the positioning of the peptide nanopackage within the loop and the wash out were optimized for gradient turnover and throughput, otherwise the peptide elution profile is identical.

The author should present a detailed explanation of the data analysis pipeline, especially regarding DIA data analysis data. Clearly specify all the softwares and packages used including versions. For example, the authors state that Spectronaut output has been used for the quantitative fragment ion profiles, however no mention of use of Spectronaut has been mentioned prior to that (the authors indicated MsFragger for library generation and DIANN for data analysis). Has this software been used for analysis or has the data been imported only to generate this specific output?

We are sorry for this confusion. Indeed, we only used MS-FRAGGER for library generation and DIA-NN 1.8 for the analysis of the single-cell and other DIA data. The references to Spectronaut in some of the subfigures is a residual from a previous manuscript version where we have actually used Spectronaut 15 for data processing. We adjusted this in the revised version within the methods section in detail.

It will also be very important to clearly specify the availability of reagents, consumables and equipment used. Part numbers and specific names of the instruments used should be reported. For example, are the EvoTips used the standard, commercially available ones?

We added this information to the manuscript within the format that MSB asked for. Indeed, we were using the standard EvoTips, which are commercially available. Within the last months EvoSep has developed a novel set of EvoTips called high-sensitivity or low retention EvoTips, which promise an improved recovery of the peptides from the EvoTip, but those have not been used in this paper.

Minor points

Please refrain from using the same colors for different parameters in the same figure as this can confuse readers, e.g., Fig 2 where the colors stand for a comparison between instruments in Fig 2A, for +/- match between run in Fig 2B and 1 vs 6 cells in Fig 2E,F.

Thank you for this observation, but subfigures are clearly separated and highlighted. As soon as we add too many colors, things might become confusing for others. This is why we would like to stay with the used color scheme that is in harmony including blue, orange and gray.

Figure 3 A mentions StageTips, which is of course the basis of EvoTips, but might be confusing since in fact EvoTips were used here.

Indeed, we call EvoTips a StageTip in the manuscript – This is because EvoTips embody the same concept as StageTips and we believe it is more scientific to refer to StageTips for that reason. However, for the reasons the reviewer mentions, we now clearly refer to Evotips where needed for access to consumables.

Please include a summary in a tabular format of the filtration steps applied to the data and the number of experiments, single cells, unique peptides, and unique proteins remaining in the dataset after each filtering step.

We added this for the protein level into the methods section.

Was the measurement of single cells randomized? Can the author at least comment on the potential acquisition biases and batch effects? For example, in Extended Figure 2B the authors present data from 3 cell culture batches that clearly separate in the PCA space. Can the author estimate whether the variation is due to biological difference between passages or technical due to batch measurement?

The measurement of the single-cells was indeed not randomized and we now mention this in the revised manuscript. All three cell culture batches presented in figure 2B were from different passages of the same cell line that have been prepared on different days. Indeed, we cannot exclude the involvement of a technical variation that leads to separation in the PCA, but as mentioned they stem from three different passages, which inevitably also represents biological variation.

Have the author considered using a CV filtering approach at the peptide/precursor level, as described in https://genomebiology.biomedcentral.com/articles/10.1186/s13059-021-02267-5#Sec16

Indeed, besides filtering the data for at least 600 protein identifications per cell and at least 15% data completeness across rows, we filtered the data for CV of less than 0.75 on protein level for differential expression analysis.

The authors used for DIA analysis a spectral library generated from fractionated peptides from HeLa cells. Have they compared to a directDIA analysis of single cell data (for example, as implemented in Spectronaut)? What is the gain given by using a deep spectral library?

We used a very concise spectral library consisting of standard, 50 ng HeLa single-shot analyses. We did not fractionate peptides to obtain a very deep library for the spectral library matching into single-cells. Since directDIA analysis methods are lacking behind even at the level of bulk analysis at this point, we did not analyze the data in directDIA mode in comparison. Still, we performed an evaluation of Spectronaut 15 for

library-based and directDIA analysis and also library-based DIA-NN analysis, which is now included into the methods section. Please see also answer to reviewer 2.

Thank you for sending us your revised manuscript. We have now heard back from reviewer #1 who was asked to evaluate your revised study. As you will see below, the reviewer thinks that the study has improved as a result of the performed revisions. However, they still express some reservations, which do not preclude the publication of the study but would need to be addressed by editing the text and carefully rewording the related statements. In addition to performing these text changes, we would also ask you to address some editorial issues listed below.

REFEREE REPORTS

Reviewer #1:

In their revised manuscripts, the authors have addressed some of my concerns and I appreciate the extra supplementary figures exemplifying these additional analyses. In my opinion, a few items still prevent acceptance of the manuscript in its current form:

1. While the authors have improved citing previous work and placing their results in context, I find the final opening statement of "However, a technology that provides quantitatively precise and accurate MS proteomics data from true single cell-derived signals (T-SCP) and answers biological questions is still outstanding." misleading, and would like to request its removal or adaptation. Perhaps the authors were just overwhelmed with excitement, but the first part of the sentence completely fails to acknowledge the technical accomplishments of previous groups in being able to successfully measure proteomes from single cells, whereas the second part discredits all biological relevance from all previous single cell proteomics studies. Moreover, the latter part suggests that this work is the first to answer biological questions, which I would argue is a bit of a reach; while 40 cells per day is a real accomplishment and significant improvement, it is still a long way off from being able to do massive, multi-1000 single cell studies as we see in the scRNAseq field. Finally, as discussed next, there is no assessment of quantitative accuracy, hence no hard claims about this can be made in the current state of the manuscript.

2. Page 5: Although the authors, in their rebuttal, claim to now have fixed the confusion surrounding quantitative accuracy & precision, they still refer to Fig 2C & D as representing accuracy. This is incorrect and needs to be fixed. As requested previously, we would like to see the measurements of quantitative accuracy, but no experiments to this end are provided. Instead, this is argued away in the rebuttal, but without mentioning appropriate citations, so this reviewer is still not convinced. If the editor feels that additional measurements are out of the scope of this manuscript, then the aspect of accuracy needs to be removed entirely (and discussed appropriately).

3. Page 13: "While single-cell transcript expression levels correlate well between the scRNA-seq technologies, they diverge from single-cell protein measurements (Fig. 5C). (Note that we performed our comparison directly on the MS intensity levels to prevent a ranking bias of proteins by their absolute abundance when using alternative quantitative approaches like iBAQ for absolute abundance representation.)"

- This might have been a misunderstanding. All intensity based global quantification methods have this bias due to differences in ionization efficiencies. The use of iBAQ or intensity is both fine, as long they mention the ranking bias.

4. Page 16: "For protein expression measurements, coefficients of variation were very small across covered abundances, independent of the data completeness (Fig. 5E, EV 5A).

- I would expect a scatterplot of data-completeness vs. CV here. I do not understand how each of the shown figures supports their claims.

5. Similar to reviewer #2, further details regarding the single cell spectral library should be provided in the manuscript, as it is currently unclear from the manuscript how this was generated. It was nice to see the Spectronaut vs DIA-NN confusion to have been clarified, thank you.

Point-by-point answers to Reviewer 1

Reviewer #1:

In their revised manuscripts, the authors have addressed some of my concerns and I appreciate the extra supplementary figures exemplifying these additional analyses. In my opinion, a few items still prevent acceptance of the manuscript in its current form:

1. While the authors have improved citing previous work and placing their results in context, I find the final opening statement of "However, a technology that provides quantitatively precise and accurate MS proteomics data from true single cell-derived signals (T-SCP) and answers biological questions is still outstanding." misleading, and would like to request its removal or adaptation. Perhaps the authors were just overwhelmed with excitement, but the first part of the sentence completely fails to acknowledge the technical accomplishments of previous groups in being able to successfully measure proteomes from single cells, whereas the second part discredits all biological relevance from all previous single cell proteomics studies. Moreover, the latter part suggests that this work is the first to answer biological questions, which I would argue is a bit of a reach; while 40 cells per day is a real accomplishment and significant improvement, it is still a long way off from being able to do massive, multi-1000 single cell studies as we see in the scRNAseq field. Finally, as discussed next, there is no assessment of quantitative accuracy, hence no hard claims about this can be made in the current state of the manuscript.

We understand and appreciate the reviewer's sentiment. We also agree that it is better to side step the question of what was and was not achieved by the previous efforts. However, it is technically correct and important to distinguish between single cell proteomics methods that actually measure single cells (requiring much more sensitive works flows) and those that obtain single cell information from multiplexing, where at least a handful of cells are analyzed together. In this second revision, we have removed the offending sentence completely. Instead, we have formulated a new paragraph that describes what we were aiming to do in this study.

"Here we set out to develop an ultrasensitive MS-based workflow that would allow quantitatively precise and accurate MS proteomics data by injecting single cells one by one into the MS – which we call true single cell-derived proteomics (T-SCP). To achieve scale, robustness and community adoption, we aimed to combine technologies that could readily be made commercially available. We apply our T-SCP technology to a drug-perturbation experiment, capturing functional, dynamic responses on a single cell population."

2. Page 5: Although the authors, in their rebuttal, claim to now have fixed the confusion surrounding quantitative accuracy & precision, they still refer to Fig 2C & D as representing accuracy. This is incorrect and needs to be fixed. As requested previously, we would like to see the measurements of quantitative accuracy, but no experiments to this end are provided. Instead, this is argued away in the rebuttal, but without mentioning appropriate citations, so this reviewer is still not convinced. If the editor feels that additional measurements are out of the scope of this manuscript, then the aspect of accuracy needs to be removed entirely (and discussed appropriately).

We agree with the reviewer that the description of quantitative accuracy throughout the single-cell experiments had been confusing and we are thankful for them to have pointed this out, so we could correct it in the revision, clearly improving the description of our data. However, the one case of the single-cell count experiment depicted in Fig. 2C and 2D is different: Here we obviously know how many

cells we injected for each of the runs (1, 2, 3, 4, 5, 6 cells, respectively). Hence these are the quantitative ratios that would be expected and that we indeed observe. So in this case our results are quantitative and from the replicates we additionally know that they are precise, which we point out in this second revision.

3. Page 13: "While single-cell transcript expression levels correlate well between the scRNA-seq technologies, they diverge from single-cell protein measurements (Fig. 5C). (Note that we performed our comparison directly on the MS intensity levels to prevent a ranking bias of proteins by their absolute abundance when using alternative quantitative approaches like iBAQ for absolute abundance representation.)"

- This might have been a misunderstanding. All intensity based global quantification methods have this bias due to differences in ionization efficiencies. The use of iBAQ or intensity is both fine, as long they mention the ranking bias.

Thank you for pointing this out. Both methods would indeed still have potential ranking biases. Our point was that 'intensity' should have less ranking bias than iBAQ, so we should have said 'minimize' instead of 'prevent'. However as this is a fairly technical point, we have deleted the entire sentence; the interested reader can still find the ranking scheme in the Material and methods part.

4. Page 16: "For protein expression measurements, coefficients of variation were very small across covered abundances, independent of the data completeness (Fig. 5E, EV 5A).

- I would expect a scatterplot of data-completeness vs. CV here. I do not understand how each of the shown figures supports their claims.

We agree and have deleted "independent of the data completeness".

5. Similar to reviewer #2, further details regarding the single cell spectral library should be provided in the manuscript, as it is currently unclear from the manuscript how this was generated. It was nice to see the Spectronaut vs DIA-NN confusion to have been clarified, thank you.

Yes, we agree that standardizing on DIA-NN will be much clearer for the reader. Regarding the generation of the spectral library, we indevoured to add as much detail as possible to describe the process (see Material and methods). Briefly, the spectral library was acquired on the same gradient as the single-cell runs. It consists of six saturated HeLa single-run measurements from the same HeLa pool that was subsequently used to perform the cell culture experiments for the single-cell cell cycle experiments. Acquired spectral library raw files were searched with standard settings in MS-FRAGGER for timsTOF data and spectral library generation. This library was then used in DIA-NN for the analysis of the single-cell runs keeping the protein grouping from the spectral library generated with MS-FRAGGER. As stated before, the goal was to keep the spectral library rather small to not artificially increase the possible search space for the single-cell data set. This is the conservative approach as larger libraries can inadvertently inflate identification numbers.

Thank you again for sending us your revised manuscript. We think that the performed revisions have satisfactorily addressed the few remaining concerns of the reviewer. I am pleased to inform you that your paper has been accepted for publication.

EMBO PRESS

YOU MUST COMPLETE ALL CELLS WITH A PINK BACKGROUND ➔

PLEASE NOTE THAT THIS CHECKLIST WILL BE PUBLISHED ALONGSIDE YOUR PAPER

Corresponding Author Name: Matthias Mann

Journal Submitted to: MSB

Manuscript Number: MSB-2021-10798

USEFUL LINKS FOR COMPLETING THIS FORM

http://www.antibodypedia.com
http://1degreebio.org
http://www.equator-network.org/reporting-guidelines/improving-bioscience-research-repor
http://grants.nih.gov/grants/olaw/olaw.htm
http://www.mrc.ac.uk/Ourresearch/Ethicsresearchguidance/Useofanimals/index.htm
http://ClinicalTrials.gov
http://www.consort-statement.org
http://www.consort-statement.org/checklists/view/32-consort/66-title
http://www.equator-network.org/reporting-guidelines/reporting-recommendations-for-tumo
http://datadryad.org
http://figshare.com
http://www.ncbi.nlm.nih.gov/gap
http://www.ebi.ac.uk/ega
http://biomodels.net/
http://biomodels.net/miriam/
http://jjj.biochem.sun.ac.za
https://osp.od.nih.gov/biosafety-biosecurity-and-emerging-biotechnology/
http://www.selectagents.gov/

Reporting Checklist For Life Sciences Articles (Rev. June 2017)

This checklist is used to ensure good reporting standards and to improve the reproducibility of published results. These guidelines are consistent with the Principles and Guidelines for Reporting Preclinical Research issued by the NIH in 2014. Please follow the journal's authorship guidelines in preparing your manuscript.

A- Figures
1. Data
The data shown in figures should satisfy the following conditions:

- ➔ the data were obtained and processed according to the field's best practice and are presented to reflect the results of the experiments in an accurate and unbiased manner.
- ➔ figure panels include only data points, measurements or observations that can be compared to each other in a scientifically meaningful way.
- ➔ graphs include clearly labeled error bars for independent experiments and sample sizes. Unless justified, error bars should not be shown for technical replicates.
- ➔ if n< 5, the individual data points from each experiment should be plotted and any statistical test employed should be justified
- ➔ Source Data should be included to report the data underlying graphs. Please follow the guidelines set out in the author ship guidelines on Data Presentation.

2. Captions
Each figure caption should contain the following information, for each panel where they are relevant:

- ➔ a specification of the experimental system investigated (eg cell line, species name).
- ➔ the assay(s) and method(s) used to carry out the reported observations and measurements
- ➔ an explicit mention of the biological and chemical entity(ies) that are being measured.
- ➔ an explicit mention of the biological and chemical entity(ies) that are altered/varied/perturbed in a controlled manner.

- ➔ the exact sample size (n) for each experimental group/condition, given as a number, not a range;
- ➔ a description of the sample collection allowing the reader to understand whether the samples represent technical or biological replicates (including how many animals, litters, cultures, etc.).
- ➔ a statement of how many times the experiment shown was independently replicated in the laboratory.
- ➔ definitions of statistical methods and measures:
  - common tests, such as t-test (please specify whether paired vs. unpaired), simple χ2 tests, Wilcoxon and Mann-Whitney tests, can be unambiguously identified by name only, but more complex techniques should be described in the methods section;
  - are tests one-sided or two-sided?
  - are there adjustments for multiple comparisons?
  - exact statistical test results, e.g., P values = x but not P values < x;
  - definition of 'center values' as median or average;
  - definition of error bars as s.d. or s.e.m.

Any descriptions too long for the figure legend should be included in the methods section and/or with the source data.

In the pink boxes below, please ensure that the answers to the following questions are reported in the manuscript itself. Every question should be answered. If the question is not relevant to your research, please write NA (non applicable). We encourage you to include a specific subsection in the methods section for statistics, reagents, animal models and human subjects.

B- Statistics and general methods

Please fill out these boxes ➔ (Do not worry if you cannot see all your text once you press return)

1.a. How was the sample size chosen to ensure adequate power to detect a pre-specified effect size?	430 single cells measured without predefined effect size. Filtering for more than 600 protein groups and 15% data completeness and CV below 0.75 as indicated in the manuscript.
1.b. For animal studies, include a statement about sample size estimate even if no statistical methods were used.	N/A
2. Describe inclusion/exclusion criteria if samples or animals were excluded from the analysis. Were the criteria pre-established?	N/A
3. Were any steps taken to minimize the effects of subjective bias when allocating animals/samples to treatment (e.g. randomization procedure)? If yes, please describe.	N/A
For animal studies, include a statement about randomization even if no randomization was used.	N/A
4.a. Were any steps taken to minimize the effects of subjective bias during group allocation or/and when assessing results (e.g. blinding of the investigator)? If yes please describe.	No
4.b. For animal studies, include a statement about blinding even if no blinding was done	N/A
5. For every figure, are statistical tests justified as appropriate?	Yes
Do the data meet the assumptions of the tests (e.g., normal distribution)? Describe any methods used to assess it.	Yes
Is there an estimate of variation within each group of data?	Yes

Is the variance similar between the groups that are being statistically compared?	Yes

C- Reagents

6. To show that antibodies were profiled for use in the system under study (assay and species), provide a citation, catalog number and/or clone number, supplementary information or reference to an antibody validation profile. e.g., Antibodypedia (see link list at top right), 1DegreeBio (see link list at top right).	
7. Identify the source of cell lines and report if they were recently authenticated (e.g., by STR profiling) and tested for mycoplasma contamination.	

* for all hyperlinks, please see the table at the top right of the document

D- Animal Models

8. Report species, strain, gender, age of animals and genetic modification status where applicable. Please detail housing and husbandry conditions and the source of animals.	N/A
9. For experiments involving live vertebrates, include a statement of compliance with ethical regulations and identify the committee(s) approving the experiments.	N/A
10. We recommend consulting the ARRIVE guidelines (see link list at top right) (PLoS Biol. 8(6), e1000412, 2010) to ensure that other relevant aspects of animal studies are adequately reported. See author guidelines, under 'Reporting Guidelines'. See also: NIH (see link list at top right) and MRC (see link list at top right) recommendations. Please confirm compliance.	N/A

E- Human Subjects

11. Identify the committee(s) approving the study protocol.	N/A
12. Include a statement confirming that informed consent was obtained from all subjects and that the experiments conformed to the principles set out in the WMA Declaration of Helsinki and the Department of Health and Human Services Belmont Report.	N/A
13. For publication of patient photos, include a statement confirming that consent to publish was obtained.	N/A
14. Report any restrictions on the availability (and/or on the use) of human data or samples.	N/A
15. Report the clinical trial registration number (at ClinicalTrials.gov or equivalent), where applicable.	N/A
16. For phase II and III randomized controlled trials, please refer to the CONSORT flow diagram (see link list at top right) and submit the CONSORT checklist (see link list at top right) with your submission. See author guidelines, under 'Reporting Guidelines'. Please confirm you have submitted this list.	N/A
17. For tumor marker prognostic studies, we recommend that you follow the REMARK reporting guidelines (see link list at top right). See author guidelines, under 'Reporting Guidelines'. Please confirm you have followed these guidelines.	N/A

F- Data Accessibility

18: Provide a "Data Availability" section at the end of the Materials & Methods, listing the accession codes for data generated in this study and deposited in a public database (e.g. RNA-Seq data: Gene Expression Omnibus GSE39462, Proteomics data: PRIDE PXD000208 etc.) Please refer to our author guidelines for 'Data Deposition'.<br><br>Data deposition in a public repository is mandatory for:<br>a. Protein, DNA and RNA sequences<br>b. Macromolecular structures<br>c. Crystallographic data for small molecules<br>d. Functional genomics data<br>e. Proteomics and molecular interactions	Yes, via PRIDE as indicated in the manuscript
19. Deposition is strongly recommended for any datasets that are central and integral to the study; please consider the journal's data policy. If no structured public repository exists for a given data type, we encourage the provision of datasets in the manuscript as a Supplementary Document (see author guidelines under 'Expanded View' or in unstructured repositories such as Dryad (see link list at top right) or Figshare (see link list at top right).	Yes, via PRIDE as indicated in the manuscript
20. Access to human clinical and genomic datasets should be provided with as few restrictions as possible while respecting ethical obligations to the patients and relevant medical and legal issues. If practically possible and compatible with the individual consent agreement used in the study, such data should be deposited in one of the major public access-controlled repositories such as dbGAP (see link list at top right) or EGA (see link list at top right).	N/A
21. Computational models that are central and integral to a study should be shared without restrictions and provided in a machine-readable form. The relevant accession numbers or links should be provided. When possible, standardized format (SBML, CellML) should be used instead of scripts (e.g. MATLAB). Authors are strongly encouraged to follow the MIRIAM guidelines (see link list at top right) and deposit their model in a public database such as Biomodels (see link list at top right) or JWS Online (see link list at top right). If computer source code is provided with the paper, it should be deposited in a public repository or included in supplementary information.	Provided in the mansucript

G- Dual use research of concern

22. Could your study fall under dual use research restrictions? Please check biosecurity documents (see link list at top right) and list of select agents and toxins (APHIS/CDC) (see link list at top right). According to our biosecurity guidelines, provide a statement only if it could.	No
